# Breakdown of 3-(allylsulfonio)propanoates in bacteria from the *Roseobacter* group yields garlic oil constituents

**DOI:** 10.3762/bjoc.17.51

**Published:** 2021-02-26

**Authors:** Anuj Kumar Chhalodia, Jeroen S Dickschat

**Affiliations:** 1 Kekulé Institute of Organic Chemistry and Biochemistry, University of Bonn, Gerhard-Domagk-Straße 1, 53121 Bonn, Germany

**Keywords:** *Allium sativum*, allyl sulfides, 3-(dimethylsulfonio)propanoate, *Roseobacter*, volatiles

## Abstract

Two analogues of 3-(dimethylsulfonio)propanoate (DMSP), 3-(diallylsulfonio)propanoate (DAllSP), and 3-(allylmethylsulfonio)propanoate (AllMSP), were synthesized and fed to marine bacteria from the *Roseobacter* clade. These bacteria are able to degrade DMSP into dimethyl sulfide and methanethiol. The DMSP analogues were also degraded, resulting in the release of allylated sulfur volatiles known from garlic. For unknown compounds, structural suggestions were made based on their mass spectrometric fragmentation pattern and confirmed by the synthesis of reference compounds. The results of the feeding experiments allowed to conclude on the substrate tolerance of DMSP degrading enzymes in marine bacteria.

## Introduction

**Scheme 1:**
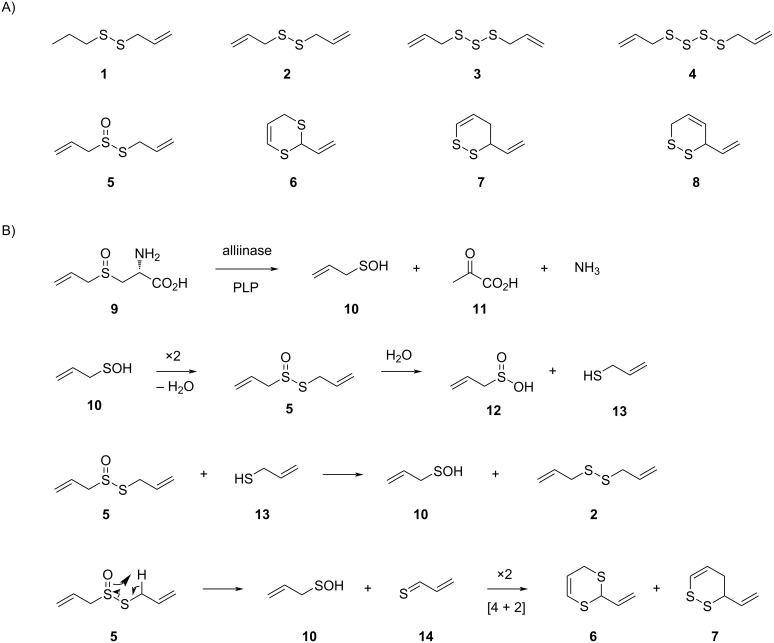
Volatile allyl sulfides. A) Compounds known from garlic oil, B) mechanism of formation from alliin (**9**) by the PLP-dependent allinase (PLP: pyridoxalphosphate) and subsequent spontaneous reactions.

The name of the allyl group has been introduced by Wertheim in 1844 when he investigated the constituents of garlic oil and derives from the botanical name of garlic (*Allium sativum*) [1]. During that time, the structures of the garlic oil constituents and also of the allyl group remained unknown, but its formula was correctly assigned as C_3_H_5_. Five decades later, Semmler reported on the nature of allyl propyl disulfide (**1**), diallyl disulfide (**2**), diallyl trisulfide (**3**), and diallyl tetrasulfide (**4**) from garlic oil (Scheme 1A) [2]. The antibacterial principle in garlic was identified in 1944 by Cavallito et al. as allicin (**5**) [3], a formal oxidation product of disulfide **2**. Not only **5**, but also several other sulfur compounds from garlic are today known to exhibit diverse biological activities, including inter alia antibacterial, antifungal, antioxidant, anti-inflammatory, and anticancer effects [4]. Later on, also heterocyclic compounds including 2-vinyl-4*H*-1,3-dithiine (**6**) and 3-vinyl-3,4-dihydro-1,2-dithiine (**7**) were discovered [5]. The formation of these volatile sulfur compounds starts from alliin (**9**) [6], a non-volatile precursor that is stored in garlic and related plants and only degraded into sulfur volatiles upon wounding by the pyridoxal phosphate (PLP) dependent alliinase (Scheme 1B) [7]. This initial enzyme-catalyzed reaction yields one equivalent of allylsulfenic acid (**10**), pyruvic acid (**11**), and ammonia from **9**, followed by a series of proposed spontaneous reactions [5,8]. Through these transformations, acid **10** can undergo a dimerization with elimination of water to allicin (**5**). The hydrolysis of **5** results in allylsulfinic acid (**12**) and allyl thiol (**13**), the latter of which can react with another molecule of **5** to yield **10** and **2**. Alternatively, **5** can decompose to **10** and thioacroleine (**14**) by a Cope elimination, which explains the formation of the heterocycles **6** and **7** by dimerization through a [4 + 2] cycloaddition [5]. Compounds **6** and **7** were also reported to be formed from **5** during gas chromatographic (GC) analysis by an unknown mechanism [9] (**7** was confused with its double bond regioisomer 3-vinyl-3,6-dihydro-1,2-dithiine (**8**) in this study [5]). Under these conditions the formation of the heterocyclic disulfides **7** and **8** may not involve a dimerization of **14**, as a [4 + 2] cycloaddition is not a preferred gas-phase reaction.

**Scheme 2:**
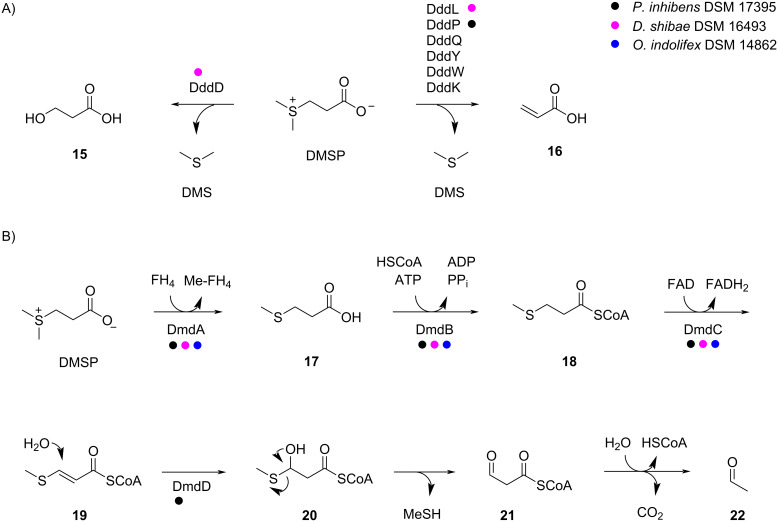
Degradation of DMSP by marine bacteria. A) Hydrolysis or lysis to DMS, B) demethylation pathway leading to MeSH. The color code shows which enzymes are encoded in the genomes of the strains investigated in this study.

The ecology of marine bacteria in their interaction with algae is particularly interesting in which the bacteria can promote the algal growth, but can also kill their host [10–11]. For both processes, the phytohormone indole-3-acetic acid is used as a messenger molecule [10]. For the macroalga *Ulva mutabilis* the presence of bacteria from the *Roseobacter* group is even mandatory for proper algal development, and 3-(dimethylsulfonio)propanoate (DMSP) is used as a chemotactic signal by the bacteria attracting them towards the algal host [12]. Many bacteria and fungi also release sulfur volatiles [13–14] that are especially important headspace constituents from marine bacteria of the *Roseobacter* group [15–17]. In these organisms, sulfur volatiles are to a large extent generated from algal (DMSP), a metabolite that is produced in massive amounts by algae [18], thus giving another example for the complex interactions between marine bacteria and algae. Known DMSP degradation pathways include its hydrolysis to dimethyl sulfide (DMS) and 3-hydroxypropanoic acid (**15**) by the enzyme DddD [19], or the lysis to DMS and acrylic acid (**16**) for which various enzymes including DddL [20], DddP [21], DddQ [22], DddY [23], DddW [24], and DddK [25] have been described (Scheme 2A). Furthermore, a demethylation pathway is known through which DMSP is first converted into methylmercaptopropanoic acid (**17**) by the tetrahydrofolate (FH_4_)-dependent demethylase, DmdA (Scheme 2B) [26]. Compound **17** can be transformed into the coenzyme A thioester **18** by the CoA ligase DmdB, followed by FAD-dependent oxidation to the α,β-unsaturated compound **19** by DmdC. The attack of water to the Michael acceptor catalyzed by the enoyl-CoA hydratase DmdD yields the hemithioacetal **20** that spontaneously collapses to methanethiol (MeSH) and malonyl-CoA semialdehyde (**21**). This compound further degrades to acetaldehyde (**22**) through the thioester hydrolysis and decarboxylation [27].

Feeding of (*methyl*-^2^H_6_)DMSP to *Phaeobacter inhibens* DSM 17395 and *Ruegeria pomeroyi* DSM 15171 resulted in the efficient uptake of labelling into dimethyl disulfide (DMDS), the oxidative dimerization product from MeSH, showing the activity of the demethylation pathway in these bacteria. However, knockout of the *dmdA* gene in *R. pomeroyi* still gave a low incorporation of labelling into DMDS, suggesting the presence of another gene responsible for the demethylation activity [28]. Also the labelling from (^34^S)DMSP was efficiently incorporated into DMDS and dimethyl trisulfide (DMTS) [29]. Our previous investigations have also demonstrated that synthetic, i.e., non-natural DMSP analogues such as 3-(ethylmethyl)sulfoniopropanoate (EMSP), 3-(diethylsulfonio)propanoate (DESP), 3-(dimethylselenio)propanoate (DMSeP; this compound is also formed naturally in *Spartina alterniflora* in the presence of sodium selenate [30]), and even 3-(dimethyltellurio)propanoate (DMTeP) are converted by the demethylation pathway into ethanethiol, methaneselenol, and methanetellurol, respectively, that further react to various volatiles containing EtS, MeSe, and MeTe groups [31]. The in vitro incubations of these DMSP analogues with recombinant DddQ and DddW from *R. pomeroyi* and DddP from *P. inhibens* demonstrated that all substrate analogues can be degraded through the lysis pathway into the corresponding dialkyl chalcogenides; only DMTeP was not cleaved by DddQ [32]. Here we describe the synthesis of the new DMSP analogues 3-(allylmethylsulfonio)propanoate (AllMSP) and 3-(diallylsulfonio)propanoate (DAllSP) and their conversion into typical garlic odor constituents by marine bacteria from the *Roseobacter* group that do not naturally occur in these organisms.

## Results and Discussion

**Scheme 3:**
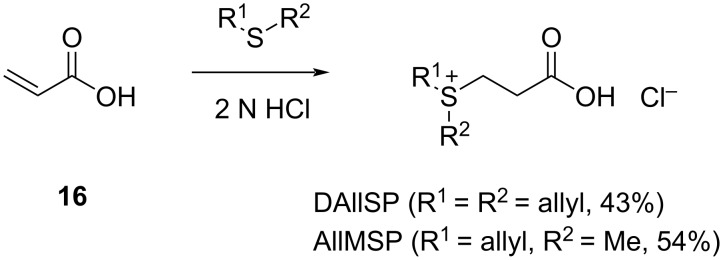
Synthesis of DMSP derivatives.

3-(Diallylsulfonio)propanoate (DAllSP) and 3-(allylmethylsulfonio)propanoate (AllMSP) were synthesized by the acid-catalyzed addition of allyl methyl sulfide and diallyl sulfide, respectively, to acrylic acid (Scheme 3). The obtained DMSP analogues were fed to marine broth agar plate cultures of three strains from the *Roseobacter* group with fully sequenced genomes, including *P. inhibens* DSM 17395, *Dinoroseobacter shibae* DSM 16493, and *Oceanibulbus indolifex* DSM 14862. In all cases the bacterial cultures released a strong garlic-like odor, presumptively due to a degradation of the DMSP derivatives to sulfur-containing volatiles, similar to the compounds known from garlic, through one of the pathways shown in Scheme 2. The emitted volatiles were captured on charcoal filter traps using a closed-loop stripping apparatus (CLSA) [33], followed by the extraction of the filters with CH_2_Cl_2_ and analysis by gas chromatography–mass spectrometry (GC–MS) of the resulting extracts. Most of the compounds were readily identified by the comparison of their mass spectra and retention indices to published data. Every experiment was performed in triplicate to check for the reproducibility of the results. For comparison, the volatiles from all three strains grown on marine broth medium without the addition of DMSP or its analogues have been reported before [31].

**Figure 1:**
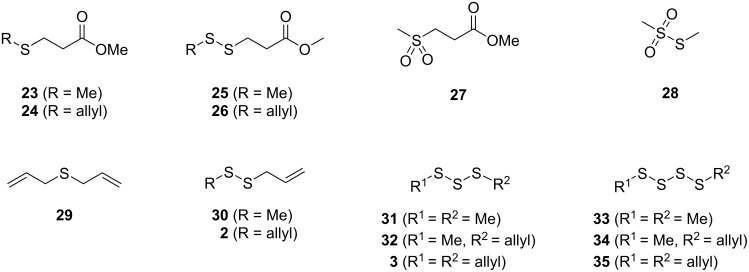
Sulfur volatiles released by agar plate cultures of marine bacteria fed with DAllSP or AllMSP.

**Figure 2:**
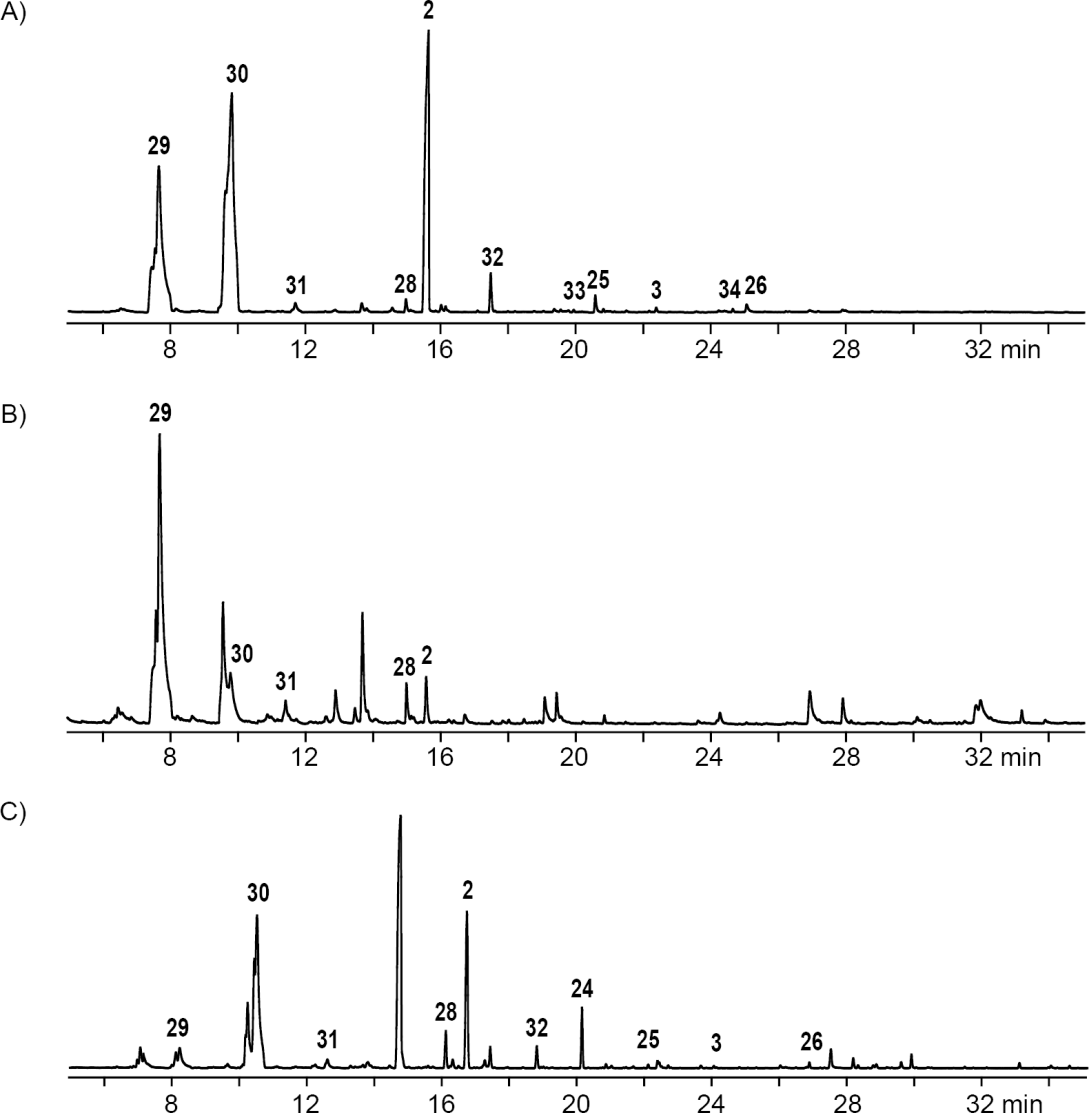
Total ion chromatograms of CLSA extracts obtained from feeding experiments with DAllSP fed to A) *P. inhibens*, B) *D. shibae*, and C) *O. indolifex*. Numbers at peaks refer to compounds in Figure 1. Peaks without numbers are unidentified.

**Scheme 4:**
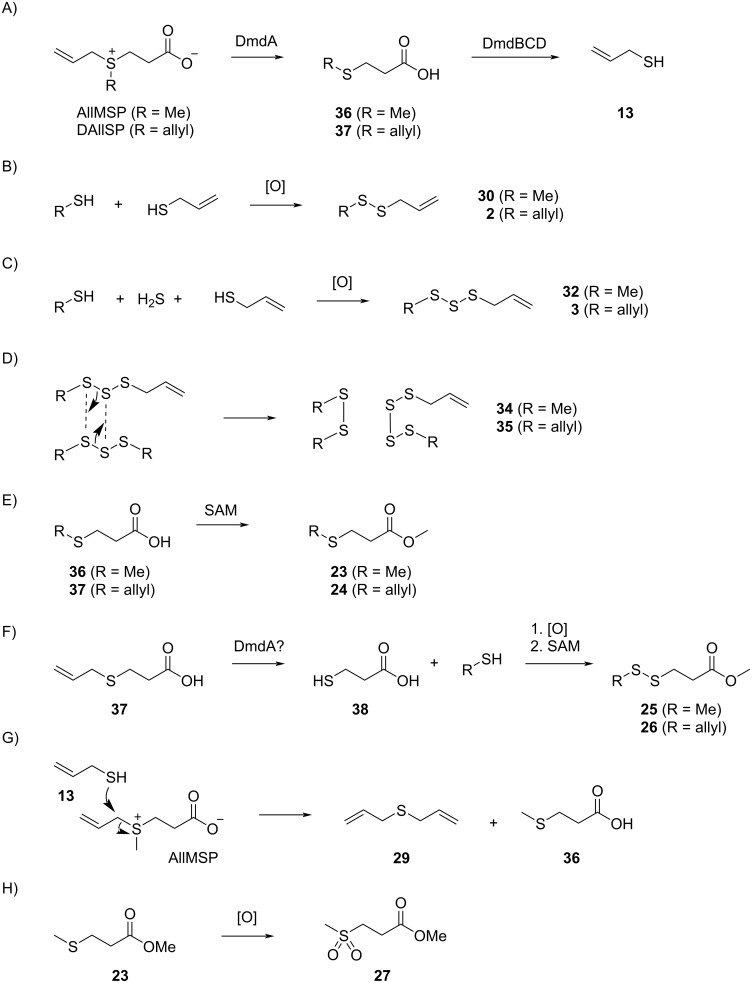
Proposed mechanisms for the formation of sulfur volatiles from DAllSP and AllMSP.

Feeding of DAllSP to *P. inhibens* resulted in the production of sulfur volatiles including several allyl derivatives (Figure 1, Figure 2A, Table 1, and Figure S1 in Supporting Information File 1). Besides the methylated sulfur compounds dimethyl trisulfide (**31**), dimethyl tetrasulfide (**33**), and *S*-methyl methanethiosulfonate (**28**) that were reported previously from *P. inhibens* [31], large amounts of diallyl sulfide (**29**) were observed, pointing to an efficient degradation of DAllSP through the lysis pathway, for which the DMSP lyase DddP can account in this organism (Scheme 2). Furthermore, the compounds allyl methyl disulfide (**30**), diallyl disulfide (**2**), allyl methyl trisulfide (**32**), and traces of diallyl trisulfide (**3**) and allyl methyl tetrasulfide (**34**) were observed. The formation of these compounds is explainable by the deallylation of DAllSP to 3-(allylsulfanyl)propanoic acid (**37**) and further degradation to allyl thiol (**13**) through the enzymes of the demethylation pathway that is fully established in *P. inhibens* by genes coding for DmdA–D (Scheme 4A). In the presence of air thiol **13** can then undergo an oxidative dimerization, or react analogously with MeSH to form allyl methyl disulfide (**30**, Scheme 4B). Similar oxidations requiring one additional unit of hydrogen sulfide can lead to the trisulfides **3** and **32** (Scheme 4C), while higher polysulfides such as **34** can arise through a metathesis reaction of two trisulfides (Scheme 4D). Also traces of methyl 3-(allylsulfanyl)propanoate (**24**), methyl 3-(methyldisulfanyl)propanoate (**25**), and methyl 3-(allyldisulfanyl)propanoate (**26**) were observed. While the presence of **24** can be explained by the *O*-methylation of the DmdA product **37** with *S*-adenosylmethionine (SAM, Scheme 4E), compounds **25** and **26** require a second deallylation of **37** to 3-mercaptopropanoic acid (**38**) possibly by DmdA, the reaction with a corresponding thiol MeSH or **13**, and *O*-methylation (Scheme 4F).

**Table 1:** Volatiles from agar plate cultures fed with DAllSP.

Compound^a^	*I*	*I*_lit._^b^	*P. in.*^c^	*D. sh.*^c^	*O. in.*^c^

diallyl sulfide (**29**)*	849	848 [34]	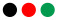	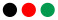	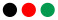
allyl methyl disulfide (**30**)	910	912 [34]	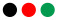	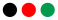	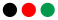
dimethyl trisulfide (**31**)*	967	970 [35]	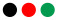	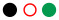	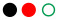
*S*-methyl methanethiosulfonate (**28**)*	1063	1068 [35]	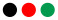	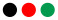	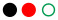
diallyl disulfide (**2**)*	1074	1075 [34]	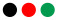	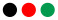	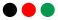
allyl methyl trisulfide (**32**)	1136	1133 [36]	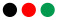	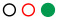	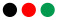
methyl 3-(allylsulfanyl)-propanoate (**24**)	1177	–	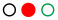	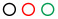	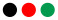
dimethyl tetrasulfide (**33**)	1216	1215 [37]	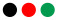	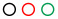	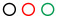
methyl 3-(methyldisulfanyl)-propanoate (**25**)*	1236	–	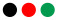	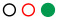	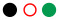
diallyl trisulfide (**3**)	1300	1300 [38]	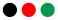	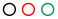	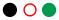
allyl methyl tetrasulfide (**34**)	1382	1371 [39]	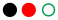	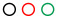	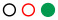
methyl 3-(allyldisulfanyl)-propanoate (**26**)*	1397	–	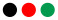	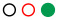	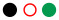
diallyl tetrasulfide (**35**)	1551	1540 [38]	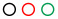	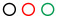	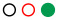

^a^Asterisks indicate the identity to a commercially available or synthetic reference standard. ^b^Retention index literature data for a HP5-MS or a similar GC column. ^c^Abbreviations are *P. in.* = *Phaeobacter inhibens*, *D. sh.* = *Dinoroseobacter shibae*, and *O. in.* = *Oceanibulbus indolifex*. Filled circles indicate the presence, non-filled circles indicate the absence of a compound in the headspace extract. The colors of the circles refer to the chromatograms in Figure 2 and Figure S1–S3 in Supporting Information File 1 with the same color.

Very similar patterns of volatiles were obtained in the feeding experiments of DAllSP with *D. shibae* and *O. indolifex* (Figure 2B,C, Table 1 and Figures S2 and S3 in Supporting Information File 1). An additionally observed compound in one analysis of *O. indolifex* was diallyl tetrasulfide (**35**). Both organisms also encode the DMSP demethylation pathway in their genomes, but with missing *dmdD* genes in both cases. A possible explanation is, that another enoyl-CoA hydratase, e.g., from fatty acid degradation, may functionally substitute for DmdD. *Dinoroseobacter shibae* additionally encodes genes for the DMSP hydrolase DddD and the DMSP lyase DddL, explaining the formation of **29**, while no DMSP hydrolase or lyase is found in *O. indolifex*. Still, compound **29** is observed within this organism, but in lower quantities than in *P. inhibens* or *D. shibae*, and may point to the presence of another, yet unidentified type of DMSP lyase in this organism, because control experiments with medium plates with DAllSP added did not show a spontaneous degradation to **29** that could explain its observation.

**Figure 3:**
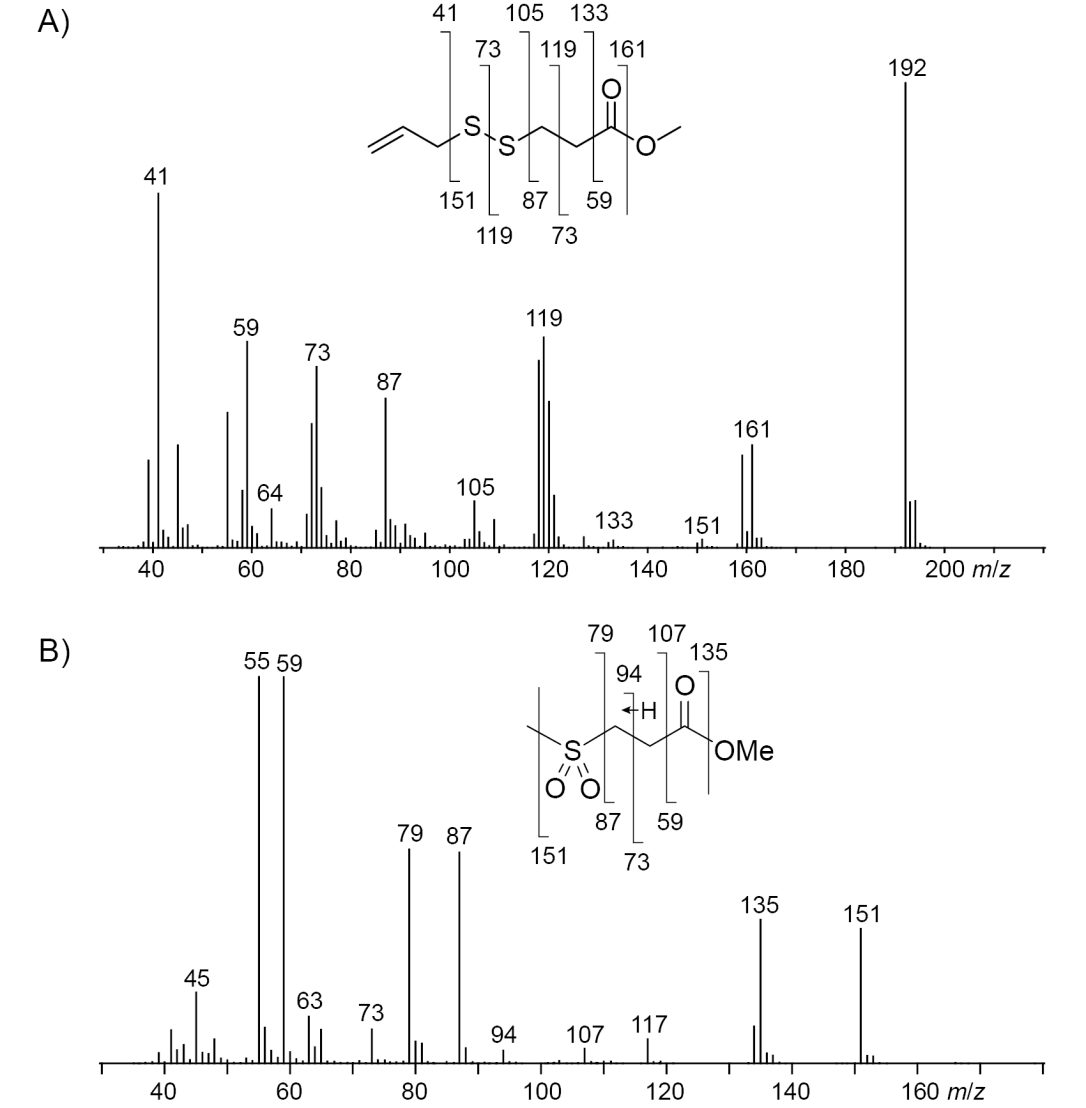
EI mass spectrum and fragmentation pattern of the unknown volatiles A) methyl 3-(allyldisulfanyl)propanoate (**26**) and B) methyl 3-(methylsulfonyl)propanoate (**27**).

**Scheme 5:**
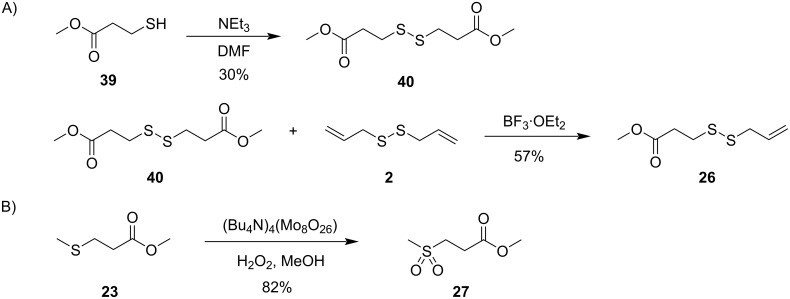
Synthesis of A) methyl 3-(allyldisulfanyl)propanoate (**26**) and B) methyl 3-(methylsulfonyl)propanoate (**27**).

The compound identification was based on a comparison to an authentic standard or of mass spectra to data base spectra in our MS libraries and confirmed for most cases by comparison of the retention indices to literature data, only for the mass spectrum of **26** no data base hit was returned. Therefore, a structural suggestion for this compound was based on the observed fragmentation pattern of the mass spectrum (Figure 3A). The molecular ion together with its isotope pattern pointed to two sulfur atoms, while the fragment ion at *m*/*z* = 64 ([S_2_]^+^) pointed to a disulfide. The fragment ions at *m*/*z* = 59 ([C_2_O_2_H_3_]^+^) and 161 ([M − OMe]^+^) indicated a methyl ester, and the series of *m*/*z* = 105 ([C_3_H_5_S_2_]^+^), 73 ([C_3_H_5_S]^+^), and 41 ([C_3_H_5_]^+^) suggested an allyl disulfide. Taken together, the structure of methyl 3-(allyldisulfanyl)propanoate was delineated for compound **26** that was further supported by additional fragmentations as shown in Figure 3A. In addition, compound **26** was synthesized by a method reported previously for the related compound **25** [40], through dimerization of methyl 3-mercaptopropanoate (**39**) to dimethyl 3,3’-disulfanediyldipropanoate (**40**), followed by the BF_3_·OEt_2_-mediated metathesis with **2** (Scheme 5A). The synthetic compound **26** was identical by mass spectrum and retention index to the unknown volatile.

**Figure 4:**
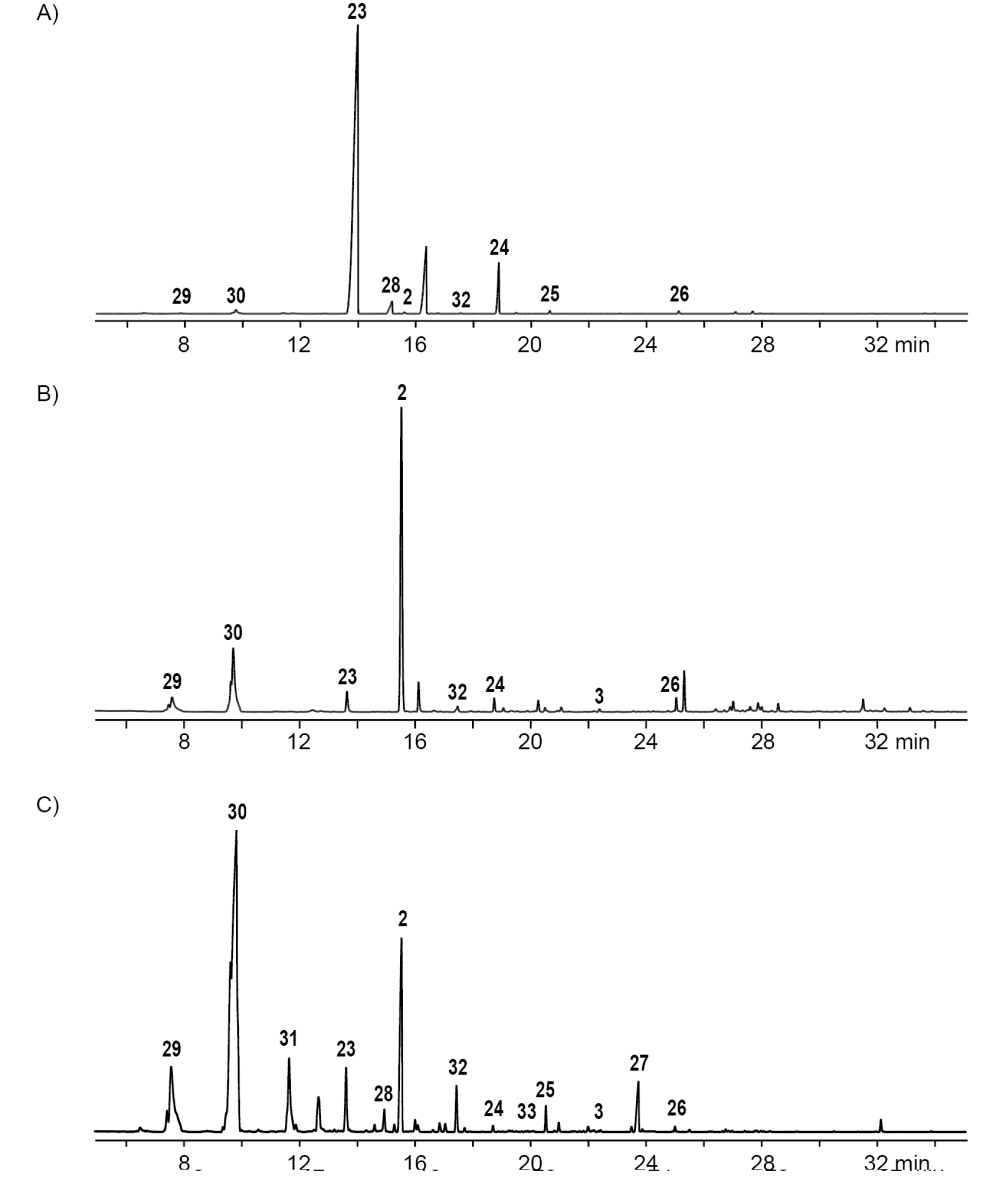
Total ion chromatograms of CLSA extracts obtained from the feeding experiments with AllMSP fed to A) *P. inhibens*, B) *D. shibae*, and C) *O. indolifex*. Numbers at peaks refer to compounds in Figure 1. Peaks without numbers are unidentified.

The feeding of AllMSP to *P. inhibens* resulted in the formation of large amounts of methyl 3-(methylsulfanyl)propanoate (**23**) in addition to smaller quantities of methyl 3-(allylsulfanyl)propanoate (**24**, Figure 4A, Table 2 and Figure S4 in Supporting Information File 1). While compound **23** can arise from AllMSP by deallylation to 3-(methylsulfanyl)propanoic acid (**36**), potentially through DmdA, and *O*-methylation, the derivative **24** may be formed analogously through intermediate **37** (Scheme 4A and E). The higher production of **23** in comparison to **24** suggests that the deallylation of AllMSP is more efficient than its demethylation, which is surprising, because naturally DmdA catalyzes a methyl-group transfer. This finding may reflect the high reactivity of the allyl group towards nucleophiles. Other compounds originating from AllMSP included the di- and trisulfides **2**, **26**, **30**, and **32** that pointed to a breakdown of AllMSP to **13** through the DMSP demethylation pathway and subsequent oxidative polysulfide formation (Scheme 4A–C), but their formation was lower than from DAllSP, likely because of the discussed efficient deallylation of AllMSP. Small amounts of diallyl sulfide (**29**) were also detected, which is the formal lysis product of DAllSP, but not of AllMSP. In first instance, its formation from AllMSP was surprising, but it is explainable by a degradation of AllMSP to **13**, followed by a nucleophilic attack at the allyl group of another AllMSP molecule (Scheme 4G). For *D. shibae* and *O. indolifex* the same pattern of compounds was found (Figure 4B,C, and Figures S5 and S6 in Supporting Information File 1), only the production of the deallylated compound **23** was lower, while in turn the production of the di- and trisulfides from **13** and of **29** was increased. This suggests that the deallylation of AllMSP by the DmdA variants in these organisms may be less efficient than was observed for *P. inhibens*. Besides these sulfur compounds, only *O. indolifex*, but not the other two strains, released another compound, **27**, whose mass spectrum was not included in our databases. The analysis of the fragmentation pattern (Figure 3B) suggested that **27** could be methyl 3-(methylsulfonyl)propanoate, an oxidation product of **23**. This hypothesis was confirmed by the chemical oxidation of **23**, yielding methyl 3-(methylsulfonyl)propanoate with an identical mass spectrum and retention index to the volatile **27** (Scheme 5B). This compound may arise from **23** by the action of an oxygenase that is restricted to *O. indolifex* and not encoded in the genomes of the other two species. Its spontaneous formation from **23** in the presence of air can be excluded, because other cultures forming **23** did not show the release of **27**.

**Table 2:** Volatiles from agar plate cultures fed with AllMSP.

Compound^a^	*I*	*I*_lit._^b^		*P. in.*^c^	*D. sh.*^c^	*O. in.*^c^

diallyl sulfide (**29**)*	849	848 [34]	1	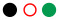	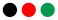	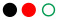
allyl methyl disulfide (**30**)	910	912 [34]	2	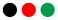	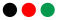	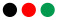
dimethyl trisulfide (**31**)*	967	970 [35]	3	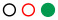	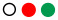	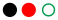
methyl 3-(methylsulfanyl)-propanoate (**23**)*	1020	1023 [41]	4	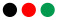	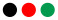	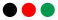
*S*-methyl methanethiosulfonate (**28**)*	1063	1068 [35]	5	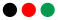	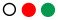	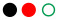
diallyl disulfide (**2**)*	1074	1075 [34]	6	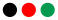	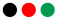	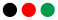
allyl methyl trisulfide (**32**)	1136	1133 [36]	7	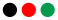	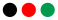	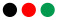
methyl 3-(allylsulfanyl)propanoate (**24**)	1177	–	8	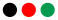	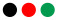	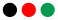
dimethyl tetrasulfide (**33**)	1216	1215 [37]	9	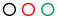	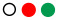	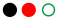
methyl 3-(methyldisulfanyl)-propanoate (**25**)*	1236	–	10	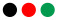	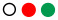	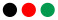
diallyl trisulfide (**3**)	1300	1300 [38]	11	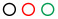	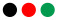	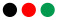
methyl 3-(methylsulfonyl)propanoate (**27**)*	1353	–	12	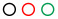	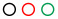	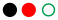
methyl 3-(allyldisulfanyl)propanoate (**26**)*	1397	–	13	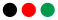	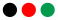	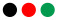

^a^Asterisks indicate the identity to a commercially available or synthetic reference standard. ^b^Retention index literature data for a HP5-MS or a similar GC column. ^c^Abbreviations are *P. in.* = *Phaeobacter inhibens*, *D. sh.* = *Dinoroseobacter shibae*, and *O. in.* = *Oceanibulbus indolifex*. Filled circles indicate the presence, non-filled circles indicate the absence of a compound in the headspace extract. The colors of the circles refer to the chromatograms in Figure 4 and Figures S4–S6 in Supporting Information File 1 with the same color.

## Conclusion

Bacteria from the *Roseobacter* group can degrade DMSP analogues with *S*-allyl groups including AllMSP and DAllSP, likely with the participation of the enzymes for DMSP (hydro)lysis and from the DMSP demethylation pathway. Because MeSH can also originate from other sources, the DMSP derivatives used in this study can lead to products that can indicate which metabolic pathways are used for their conversion. Interestingly, the volatiles formed from AllMSP and DAllSP closely resemble flavoring compounds from garlic. The demethylation pathway with all four enzymes DmdABCD is fully established in *P. inhibens*, while genes for DmdD are missing in *D. shibae* and *O. indolifex*, suggesting that another enzyme with a low sequence homology may substitute for DmdD, leading to allylthiol and several sulfur volatiles derived from it in all three strains. The DMSP hydrolase DddD and the lyase DddL are present in *D. shibae*, and *P. inhibens* has a DMSP lyase DddP, which can explain the conversion of DAllSP into diallyl sulfide, while the reason for its formation in *O. indolifex* is currently unclear and may point to an unknown type of DMSP lyase in this organism. Since the observed patterns of allylated sulfur volatiles in the three investigated strains are different, it seems possible that the DMSP (hydro)lases and the enzymes from the DMSP demethylation pathway have different activities towards AllMSP and DAllSP. In vitro studies with recombinant purified enzymes and mutational work will be needed for more detailed insights to support our hypotheses regarding the involved enzymes in AllMSP and DAllSP breakdown and will be performed in our laboratories in the future.

## Experimental

### Strains and culture condition

*Phaeobacter inhibens* DSM 14862, *Dinoroseobacter shibae* DSM 16493, *Oceanibulbus indolifex* DSM 14862 were precultured in full strength marine broth medium (MB 2216, Roth) at 28 °C with shaking at 180 rpm until the OD value reached about 1.0.

### Feeding experiments and sampling of volatiles

Headspace samplings for each strain were done in triplicates. For the feeding experiments, DAllSP or AlMSP (1 mM) were added to the full strength marine broth agar medium (MB2216) after autoclavation. The medium was then transferred into glass Petri dishes. The agar plates were inoculated with the precultures (400 μL), incubated for two days at 28 °C and then subjected for headspace extraction to a CLSA [33] for 24 h. The released volatiles were collected on charcoal filters (Chromtech, Idstein, Germany), followed by the extraction of the filters with dichloromethane (50 μL), and analysis of the extracts by GC–MS. For comparison, blank experiments with MB medium alone and with MB agar plates spiked with DAllSP or AlMSP were performed in the same way. All the volatiles mentioned in Table 1 and Table 2 were not observed in the blank experiments.

### GC–MS

The GC–MS analyses were carried out on a HP7890A GC system connected to a HP5975C mass selective detector fitted with a HP-5MS fused silica capillary column (30 m × 0.22 mm i.d., 0.25 μm film, Hewlett-Packard). The conditions were: inlet pressure: 67 kPa, He 23.3 mL min^−1^; injection volume: 1 μL; injector: 250 °C; transfer line: 300 °C; electron energy: 70 eV. The GC was programmed as follows: 50 °C (5 min isothermic), increasing at 5 °C min^−1^ to 320 °C and operated in the splitless mode (60 s valve time); carrier gas (He): 1.2 mL min^−1^. The retention indices were determined from *n*-alkane standards (C_8_–C_32_) [42].

### General synthetic methods

All chemicals were purchased from TCI (Deutschland) or Sigma-Aldrich Chemie (Germany), and used without purification. Solvents were distilled and dried by standard methods. NMR spectra were recorded on a Bruker (Billerica, USA) Avance III HD Prodigy (500 MHz) or on an Avance III HD Cryo (700 MHz) NMR spectrometer. The spectra were referenced against solvent signals (^1^H NMR, residual proton signal: D_2_O δ = 4.79 ppm, CDCl_3_ δ = 7.26 ppm, *d*_6_-DMSO δ = 2.50 ppm; ^13^C NMR: CDCl_3_ δ = 77.16 ppm, *d*_6_-DMSO δ = 39.52 ppm). The coupling constants are given in Hz. IR spectra were recorded on a Bruker α spectrometer equipped with a diamond-ATR probe. The relative intensities of signals are indicated by w (weak), m (medium), and s (strong).

### Synthesis of allyl DMSP derivatives

A mixture of acrylic acid (0.72 g, 10 mmol) and diallyl sulfide or allylmethyl sulfide (10 mmol) was treated with 2 N HCl at 80 °C for 4 h. The reaction mixture was concentrated in vacuo and the residue was purified by silica gel column chromatography (CH_2_Cl_2_/MeOH 5:1), followed by recrystallization from methanol/diethyl ether 1:1 to yield the pure compounds.

**DAllSP·HCl.** Yield: 960 mg (4.32 mmol, 43%). ^1^H NMR (D_2_O, 700 MHz) δ 5.98 (ddt, *J* = 17.5, 10.2, 7.4, 2H), 5.73 (d, *J* = 10.2, 2H), 5.72 (d, *J* = 17.0, 2H), 4.08 (d, *J* = 7.4, 4H), 3.43 (t, *J* = 6.9, 2H), 2.78 (t, *J* = 6.9, 2H); ^13^C NMR (D_2_O, 175 MHz) δ 177.05 (C), 127.65 (2 × CH), 123.54 (2 × CH_2_), 41.53 (2 × CH_2_), 35.08 (CH_2_), 31.68 (CH_2_); HRMS–EI (*m*/*z*): calcd for [C_9_H_15_O_2_S]^+^, 187.0787; found, 187.0790.

**AllMSP·HCl.** Yield: 1.06 g (5.41 mmol, 54%). ^1^H NMR (D_2_O, 700 MHz) δ 5.96 (ddt, *J* = 17.5, 10.2, 7.5, 1H), 5.74 (d, *J* = 10.2, 1H), 5.71 (d, *J* = 17.2, 1H), 4.13 (dd, *J* = 13.4, 7.4, 1H), 4.09 (dd, *J* = 13.4, 7.5, 1H), 3.58 (dt, *J* = 13.7, 6.9, 1H), 3.47 (dt, *J* = 13.5, 6.7, 1H), 3.04 (t, *J* = 6.8, 2H), 2.91 (s, 3H); ^13^C NMR (D_2_O, 175 MHz) δ 173.77 (C), 128.19 (CH), 122.74 (CH_2_), 43.82 (CH_2_), 35.84 (CH_2_), 28.75 (CH_2_), 21.72 (CH_3_); HRMS–EI (*m*/*z*): calcd for [C_7_H_13_O_2_S]^+^, 161.0631; found, 161.0630.

### Synthesis of dimethyl 3,3’-disulfanediyldipropanoate (**40**)

A solution of methyl 3-mercaptopropanoate (6.00 g, 50.0 mmol, 1.0 equiv) and triethylamine (5.05 g, 50.0 mmol) in DMF (10 mL) was treated for 24 h at 40 °C. The reaction was quenched by the addition of water and the aqueous phase extracted with ethyl acetate. The extract was dried with MgSO_4_ and then concentrated in vacuo. The residue was purified by silica column chromatography (cyclohexane/EtOAc 5:1) to give compound **40** (1.80 g, 7.56 mmol, 30%) as pale yellow oil. TLC *R*_f_ 0.44 (cyclohexane/EtOAc 10:3); IR (diamond-ATR) ν̃: 2998 (w), 2952 (w), 2845 (w), 2256 (w), 1730 (m), 1436 (w), 1354 (w), 1240 (w), 1215(w), 1195 (w), 1171 (w), 1139 (w), 1046 (w), 1017 (w), 979 (w), 907 (w), 822 (w), 726 (m), 648 (w), 435 (w) cm^−1^; ^1^H NMR (CDCl_3_, 500 MHz) δ 3.64 (s, 6H), 2.87 (t, *J* = 7.2, 4H), 2.68 (t, *J* = 7.2, 4H) ppm; ^13^C NMR (CDCl_3_, 125 MHz) δ 172.11 (2 × C), 51.90 (2 × CH_3_), 33.93 (2 × CH_2_), 33.16 (2 × CH_2_) ppm.

### Synthesis of methyl 3-(allyldisulfanyl)propanoate (**26**)

To a solution of dimethyl 3,3’-disulfanediyldipropanoate (**40**, 0.50 g, 2.10 mmol, 1.0 equiv) and diallyl disulfide (**2**, 0.31 g, 2.10 mmol, 1.0 equiv) in dry DCM (10 mL) and CH_3_NO_2_ (10 mL) at 0 °C BF_3_·OEt_2_ (30 mg, 0.21 mmol, 0.1 equiv) was added. The reaction mixture was stirred at 0 °C for 3 h and at room temperature overnight. The reaction was quenched by the addition of water and extracted with ethyl acetate. The extracts were dried with MgSO_4_ and concentrated in vacuo. The obtained residue was purified by silica gel column chromatography (cyclohexane/EtOAc 5:1) to give compound **26** (0.23 g, 1.20 mmol, 57%). TLC *R*_f_ = 0.72 (cyclohexane/EtOAc = 1:1); IR (diamond-ATR) ν̃: 3082 (w), 2950 (w), 2845 (w), 1736 (s), 1634 (w), 1435 (w), 1354 (w), 1277 (w), 1240 (w), 1216 (w), 1172 (w), 1144 (w), 1017 (w), 985 (w), 922 (w), 859 (w), 820 (w), 756 (w), 722 (w), 669 (w), 582 (w), 478 (w), 435 (w) cm^−1^; ^1^H NMR (CDCl_3_, 500 MHz) δ 5.83 (ddt, *J* = 17.1, 9.9, 7.3, 1H), 5.19 (ddt, *J* = 16.9, 1.3, 1.3, 1H), 5.14 (dm, *J* = 10.0, 1H), 3.69 (s, 3H), 3.32 (dm, *J* = 7.3, 2H), 2.91 (t, *J* = 7.2, 2H), 2.72 (t, *J* = 7.2, 2H) ppm; ^13^C NMR (CDCl_3_, 125 MHz) δ 172.14 (C), 132.71 (CH), 119.40 (CH_2_), 52.04 (CH_3_), 41.60 (CH_2_), 33.87 (CH_2_), 33.40 (CH_2_) ppm; HRMS–EI (*m*/*z*): calcd for [C_7_H_12_O_2_S_2_]^+^, 192.0273; found, 192.0289.

### Synthesis of methyl 3-(methylsulfonyl)propanoate (**27**)

To a stirred solution of [(*n*-C_4_H_9_)_4_N]_4_(Mo_8_O_26_) (5 mg, 2.5 μmol, 0.001 equiv) [43] in methanol (4 mL), methyl 3-methylthiopropanoate (335 mg, 2.50 mmol, 1.0 equiv) was added at 40 °C. After the reaction mixture was stirred for 5 minutes, 30% hydrogen peroxide solution (0.52 mL, 0.57 g, 5.0 mmol, 2.0 equiv) was added dropwise. The color of the reaction mixture changed from colorless to yellow. The reaction mixture was stirred for 30 minutes at room temperature. After completion of the reaction, EtOAc (10 mL) was added, causing precipitation of the catalyst. The catalyst was filtered off, the filtrate was dried with MgSO_4_ and concentrated in vacuo to give pure **27** (0.34 g, 2.05 mmol, 82%) as colorless solid. TLC *R*_f_ 0.17 (cyclohexane/EtOAc 1:1); IR (diamond-ATR) ν̃: 3014 (w), 2948 (w), 2932 (w), 1762 (m), 1687 (w), 1442 (w), 1433 (w), 1418 (w), 1375 (w), 1331 (w), 1306 (m), 1373 (m), 1259 (m), 1203 (w), 1180 (w), 1131 (m), 1056 (w), 1004 (w), 988 (w), 971 (w), 956 (w), 898 (w), 786 (w), 774 (w), 749 (w), 601 (w), 514 (w), 505 (w), 441 (w) cm^−1^; ^1^H NMR (*d*_6_-DMSO, 500 MHz) δ 3.63 (s, 3H), 3.37 (t, *J* = 7.5, 2H), 3.01 (s, 3H), 2.78 (t, *J* = 7.5, 2H) ppm; ^13^C NMR (*d*_6_-DMSO, 125 MHz) δ 170.79 (C), 51.88 (CH_3_), 49.14 (CH_2_), 40.21 (CH_3_), 26.89 (CH_2_) ppm.

## Supporting Information

File 1 Additional total ion chromatograms and copies of NMR spectra.
