# Supplementary material for: Breakdown of 3-(allylsulfonio)propanoates in bacteria from the Roseobacter group yields garlic oil constituents
Source: Beilstein J Org Chem. 2021 Feb 26;17:569–80. doi: 10.3762/bjoc.17.51 (PMC7934745; doi:10.3762/bjoc.17.51)
Supplement: File 1 — Additional total ion chromatograms and copies of NMR spectra. [file Beilstein_J_Org_Chem-17-569-s001.pdf]

BEILSTEIN JOURNAL OF ORGANIC CHEMISTRY

Supporting Information

for

Breakdown of 3-(allylsulfonio)propanoates in bacteria from the *Roseobacter* group yields garlic oil constituents

Anuj Kumar Chhalodia and Jeroen S. Dickschat

Beilstein J. Org. Chem. **2021,** *17,* 569–580. doi:10.3762/bjoc.17.51

Additional total ion chromatograms and copies of NMR spectra

License and Terms: This is a supporting information file under the terms of the Creative Commons Attribution License (https://creativecommons.org/licenses/by/4.0). Please note that the reuse, redistribution and reproduction in particular requires that the author(s) and source are credited and that individual graphics may be subject to special legal provisions.
The license is subject to the *Beilstein Journal of Organic Chemistry* terms and conditions: (https://www.beilstein-journals.org/bjoc/terms)

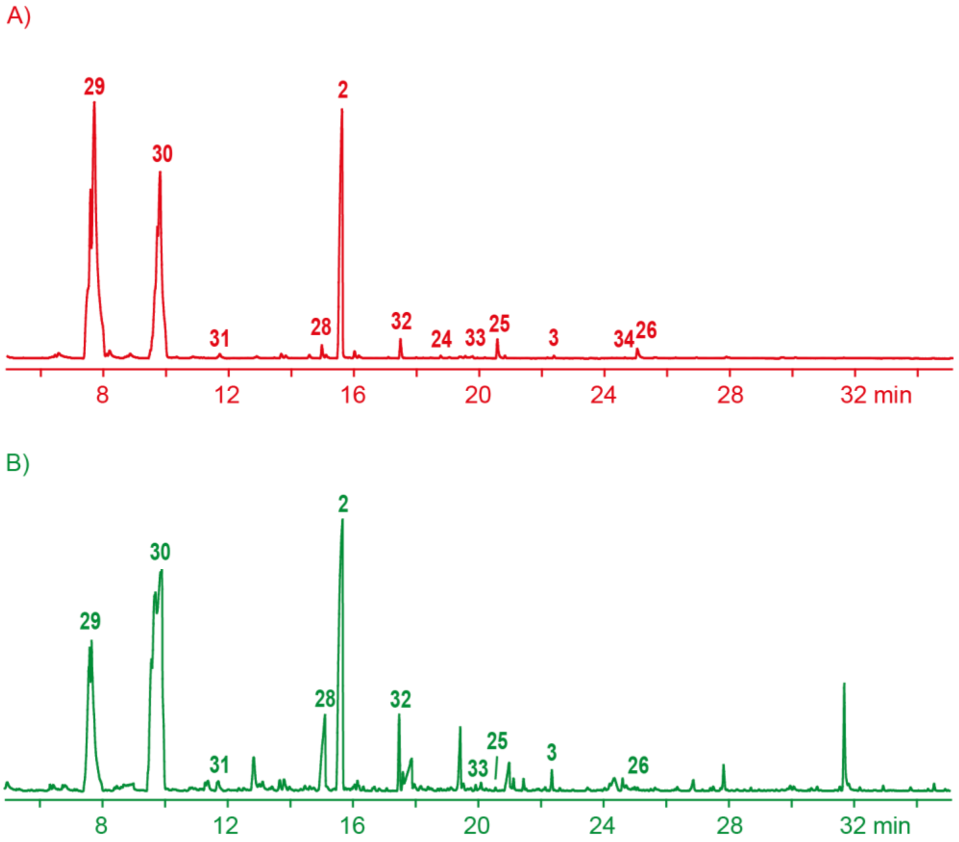

Figure S1: Total ion chromatograms of CLSA extracts obtained from feeding experiments with DAllSP fed to *P. inhibens* (two repetitions of experiment shown in Figure 2A of main text).

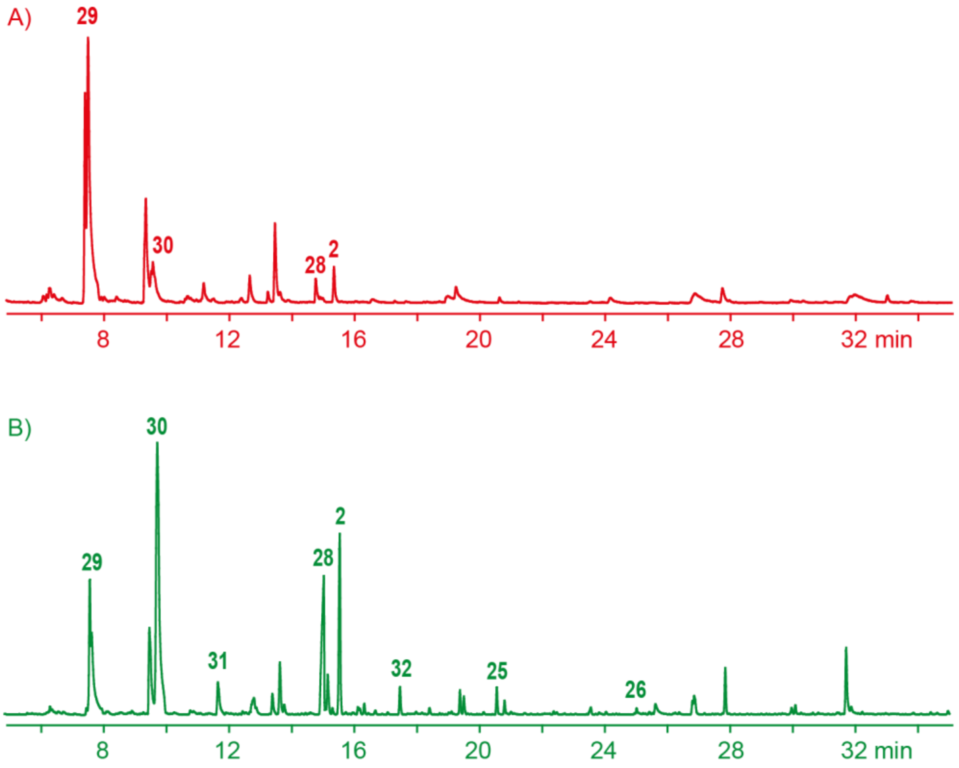

Figure S2: Total ion chromatograms of CLSA extracts obtained from feeding experiments with DAllSP fed to *D. shibae* (two repetitions of experiment shown in Figure 2B of main text).

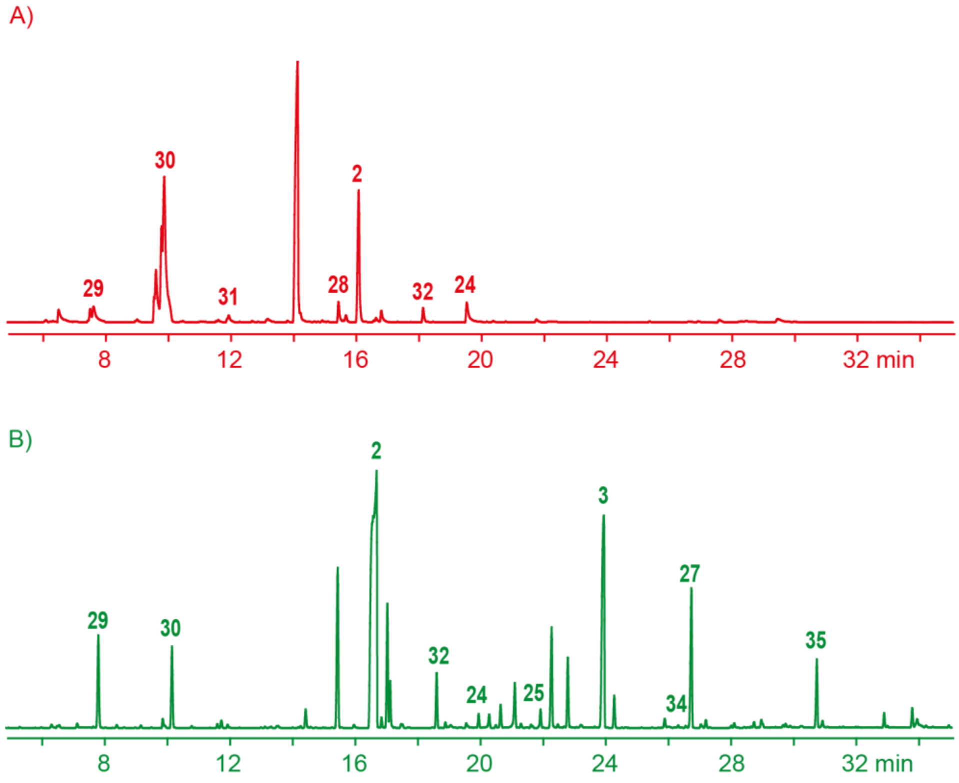

Figure S3: Total ion chromatograms of CLSA extracts obtained from feeding experiments with DAllSP fed to *O. indolifex* (two repetitions of experiment shown in Figure 2C of main text).

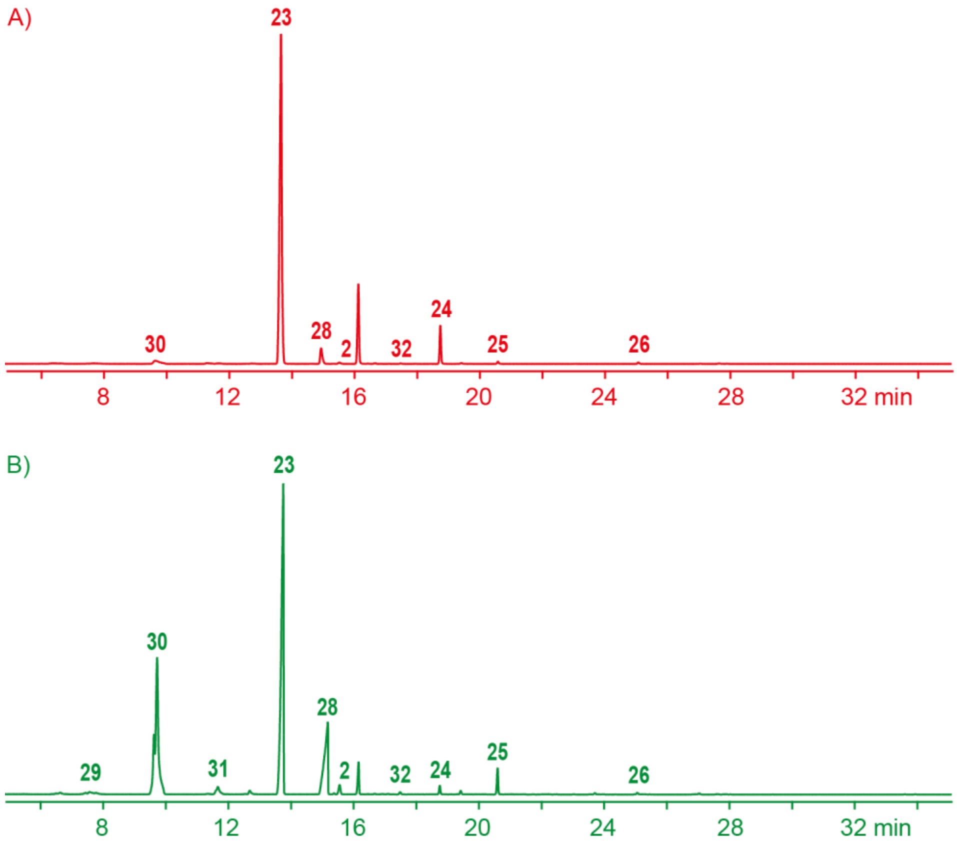

Figure S4: Total ion chromatograms of CLSA extracts obtained from feeding experiments with AllMSP fed to *P. inhibens* (two repetitions of experiment shown in Figure 4A of main text).

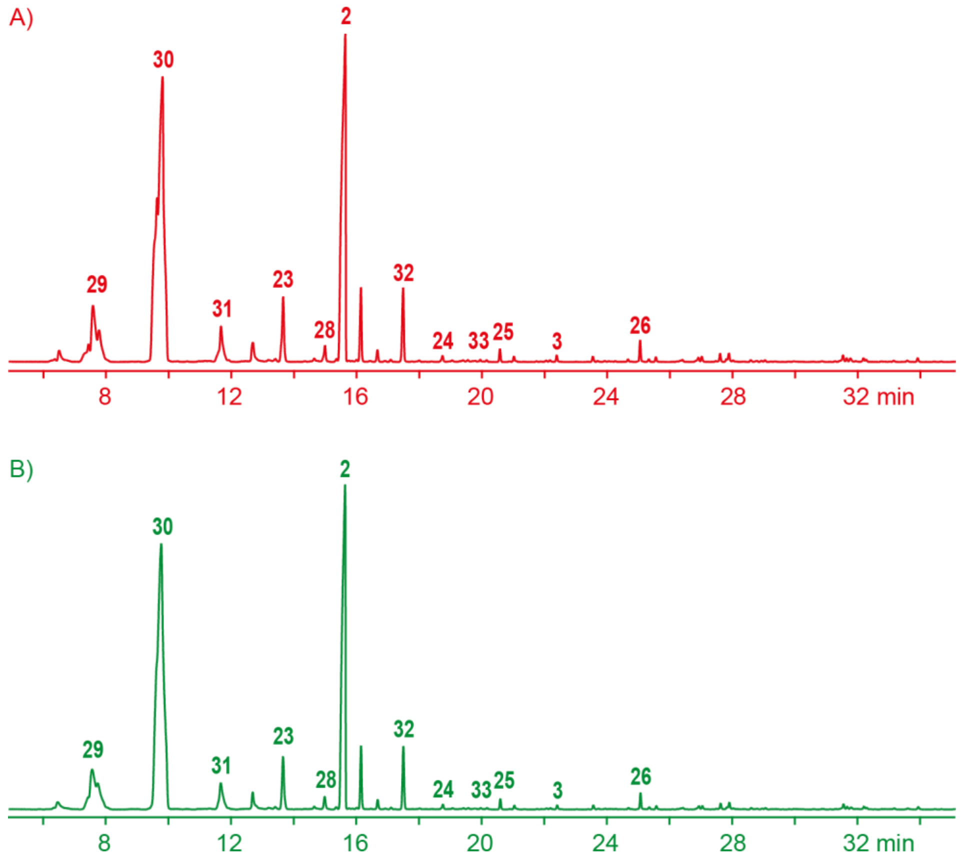

Figure S5: Total ion chromatograms of CLSA extracts obtained from feeding experiments with AllMSP fed to *D. shibae* (two repetitions of experiment shown in Figure 4B of main text).

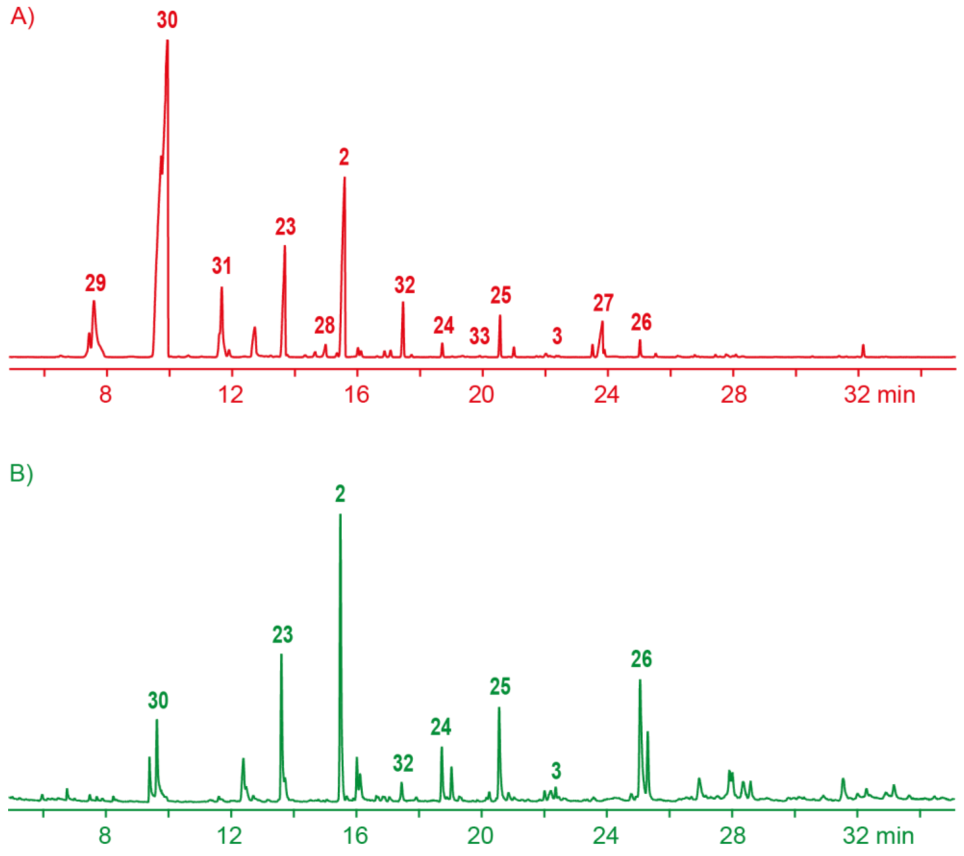

Figure S6: Total ion chromatograms of CLSA extracts obtained from feeding experiments with AllMSP fed to *O. indolifex* (two repetitions of experiment shown in Figure 4C of main text).

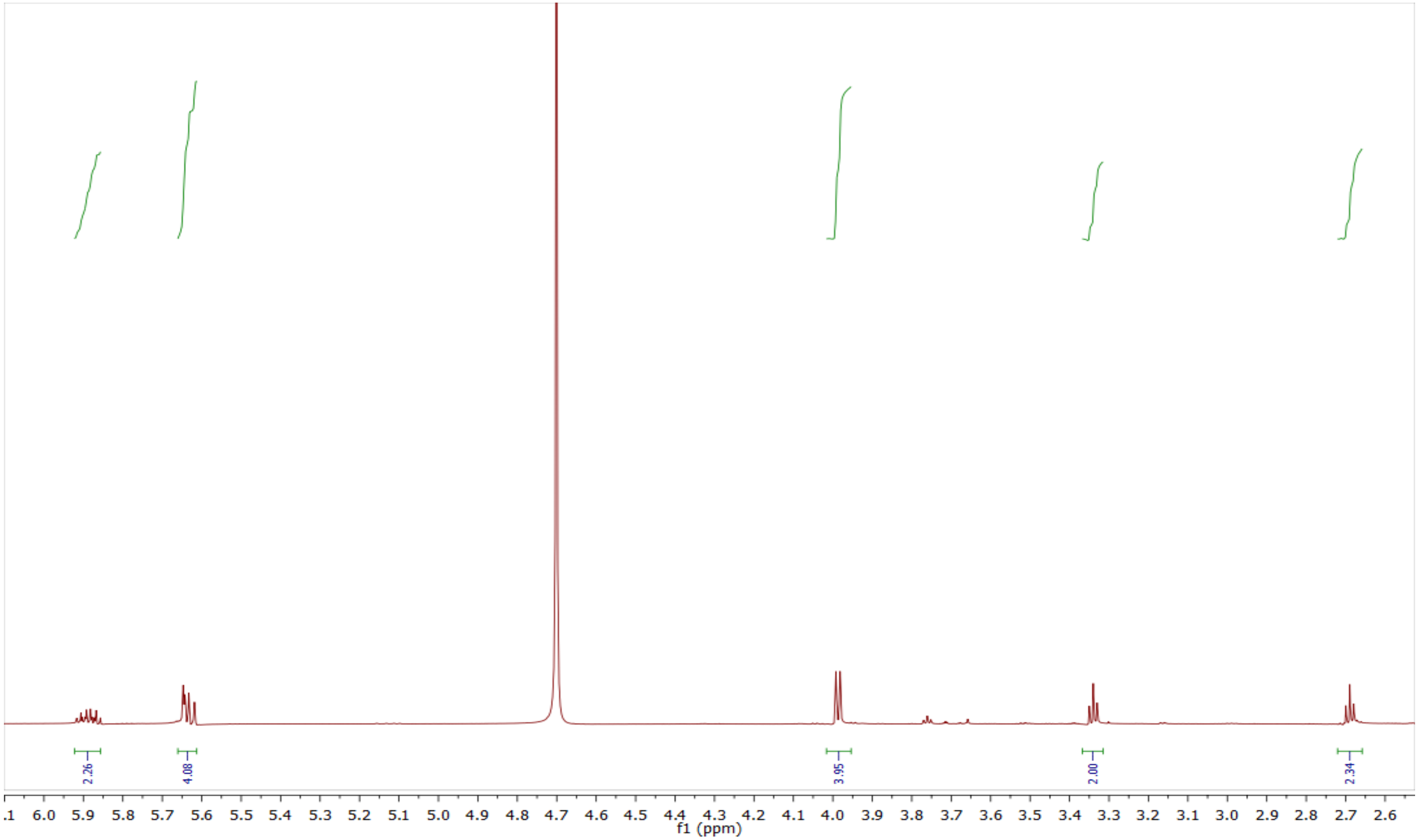

Figure S7: ^{1}H NMR spectrum (D_2O, 700 MHz) of DAllSP.

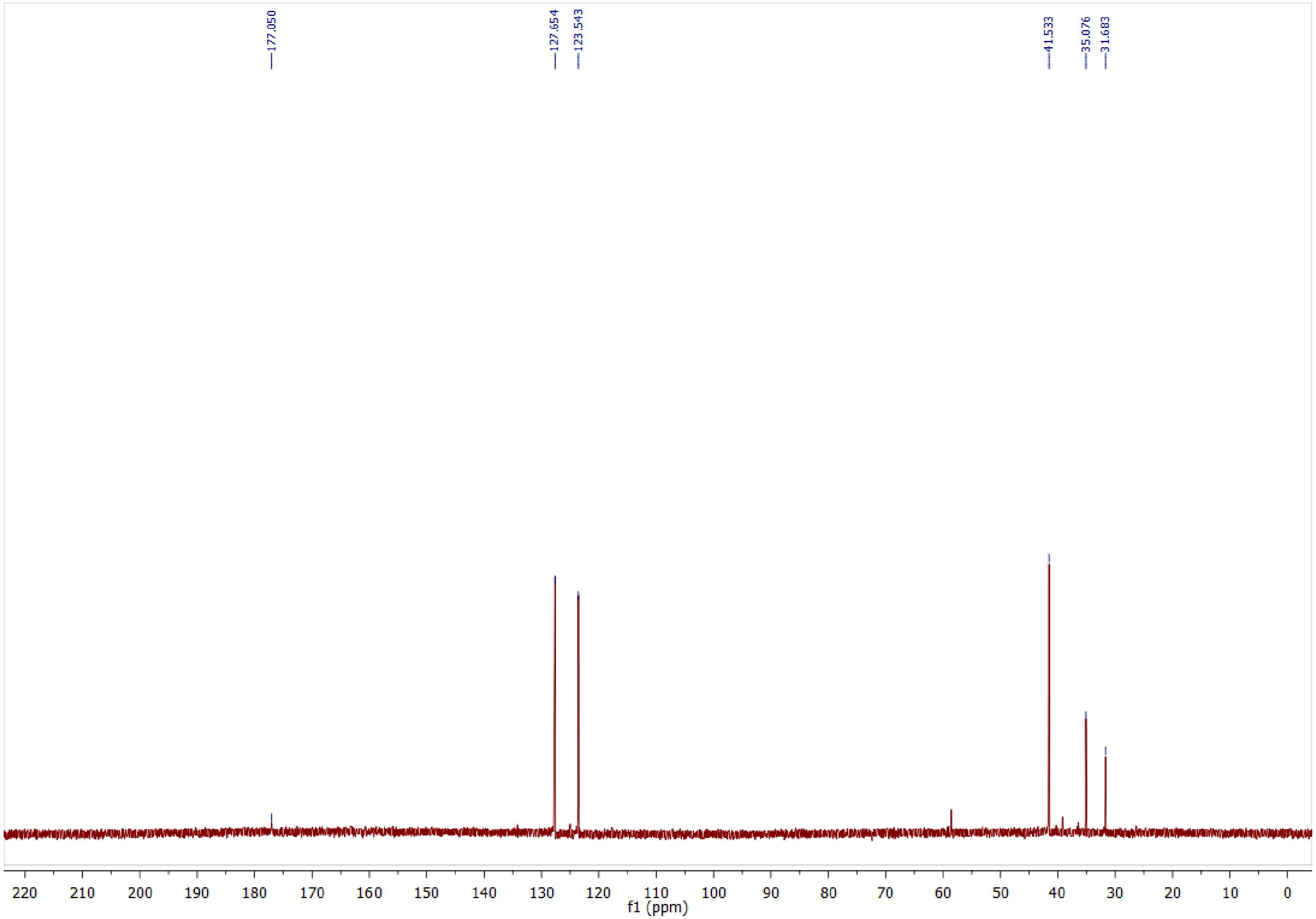

Figure S8: ^{13}C NMR spectrum (D_2O, 175 MHz) of DAllSP.

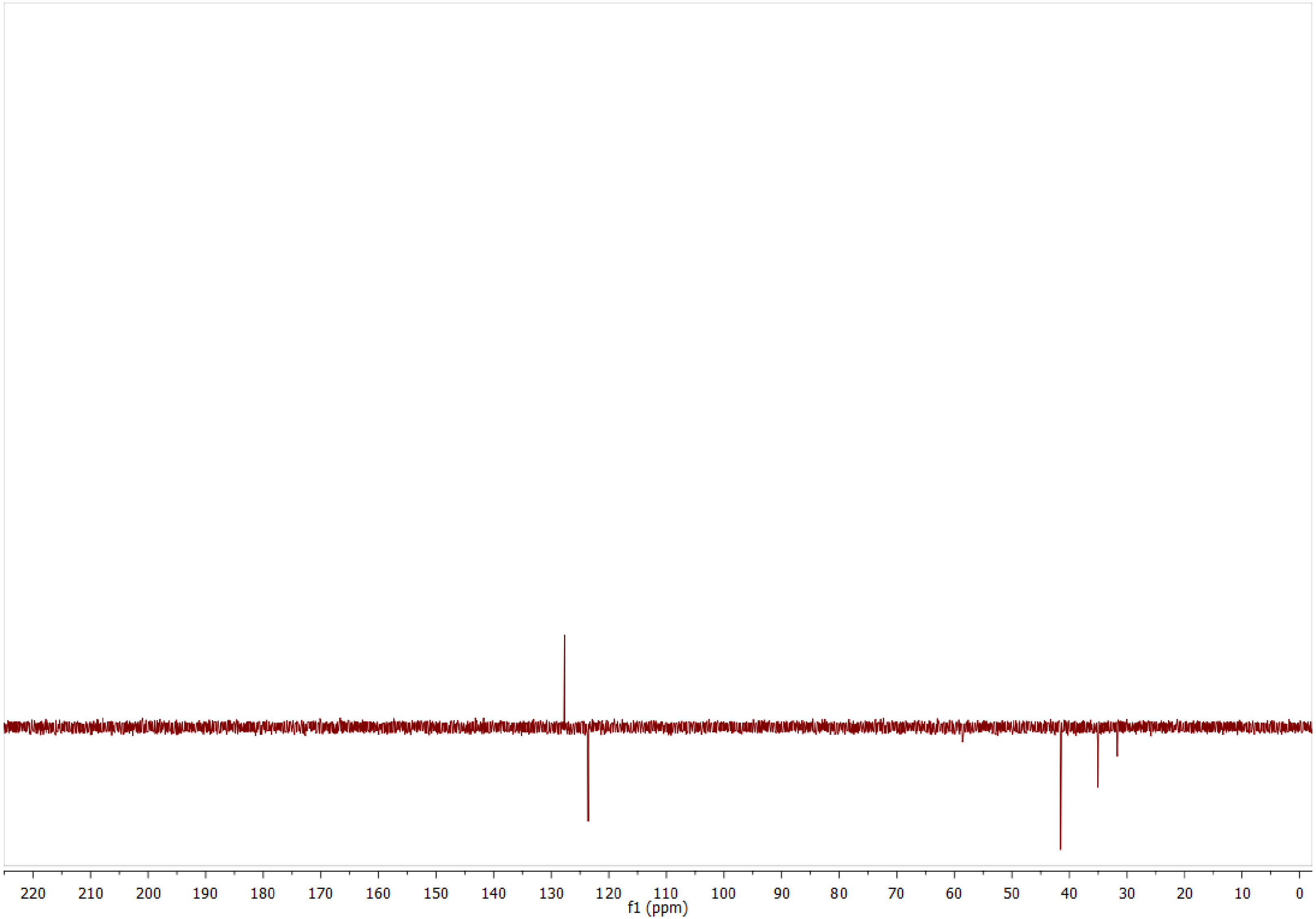

Figure S9: ^{13}C-DEPT spectrum (D_2O, 175 MHz) of DAllSP.

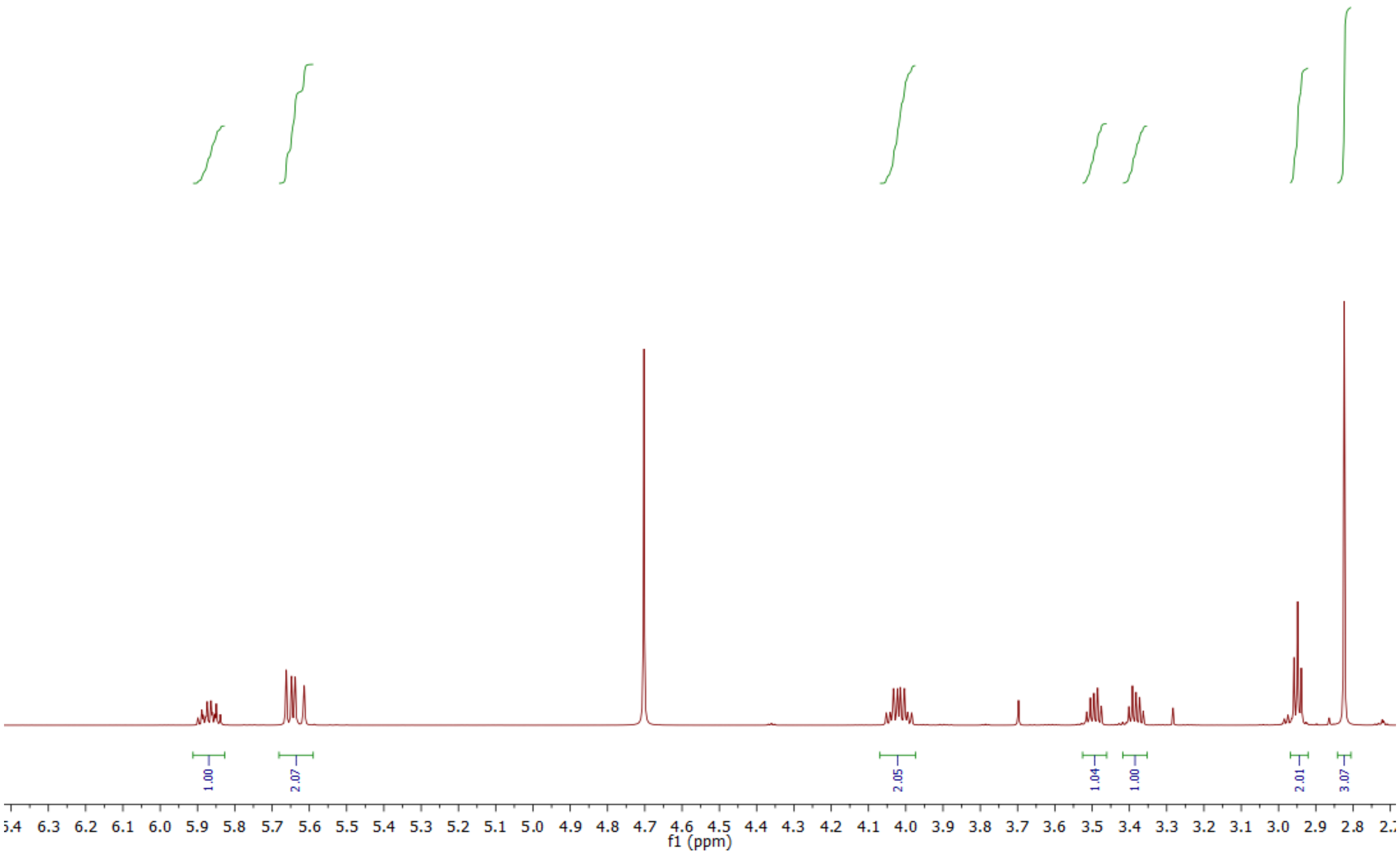

Figure S10: ^{1}H NMR spectrum (D_2O, 700 MHz) of AllMSP.

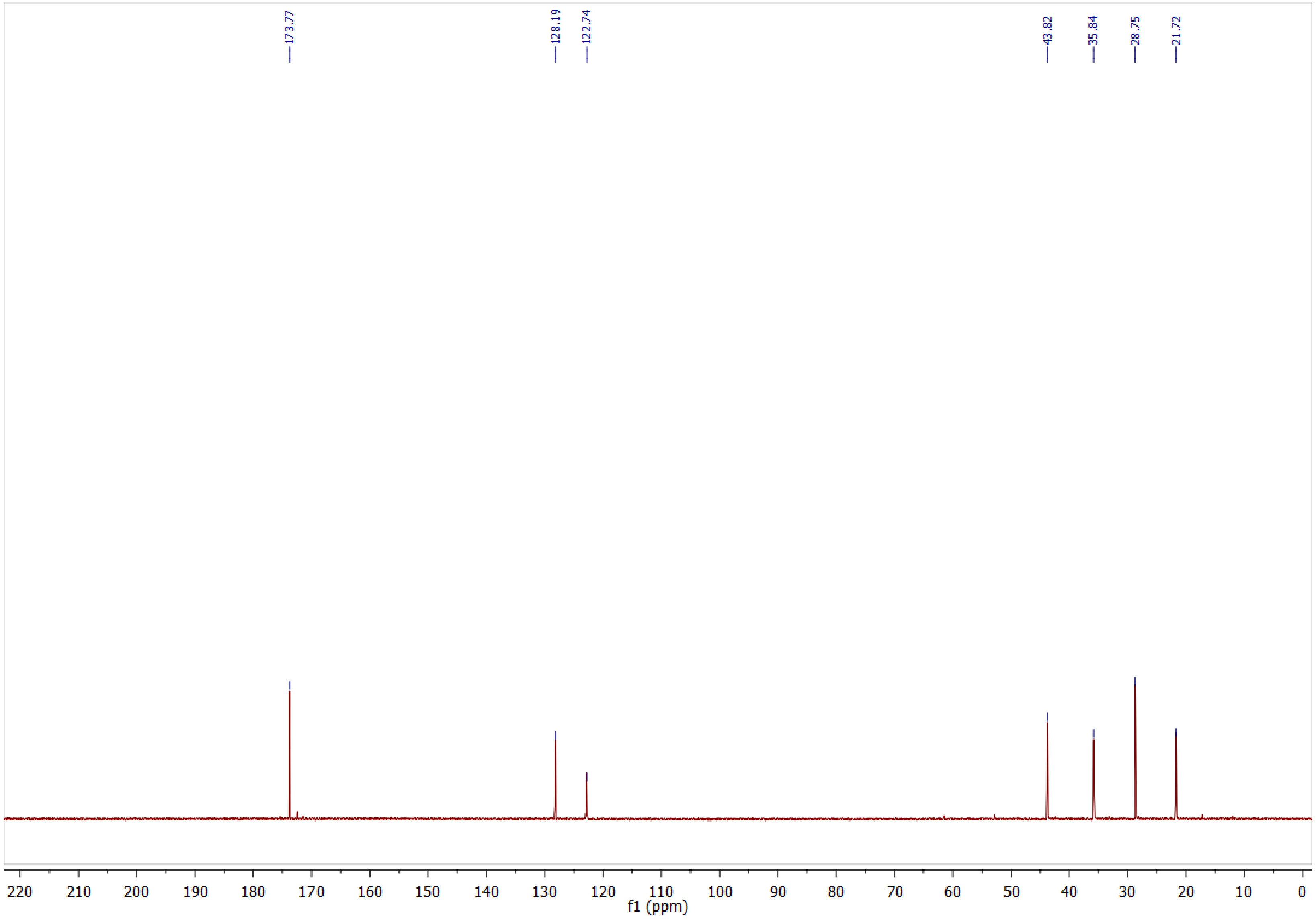

Figure S11: ^{13}C NMR spectrum (D_2O, 175 MHz) of AllMSP.

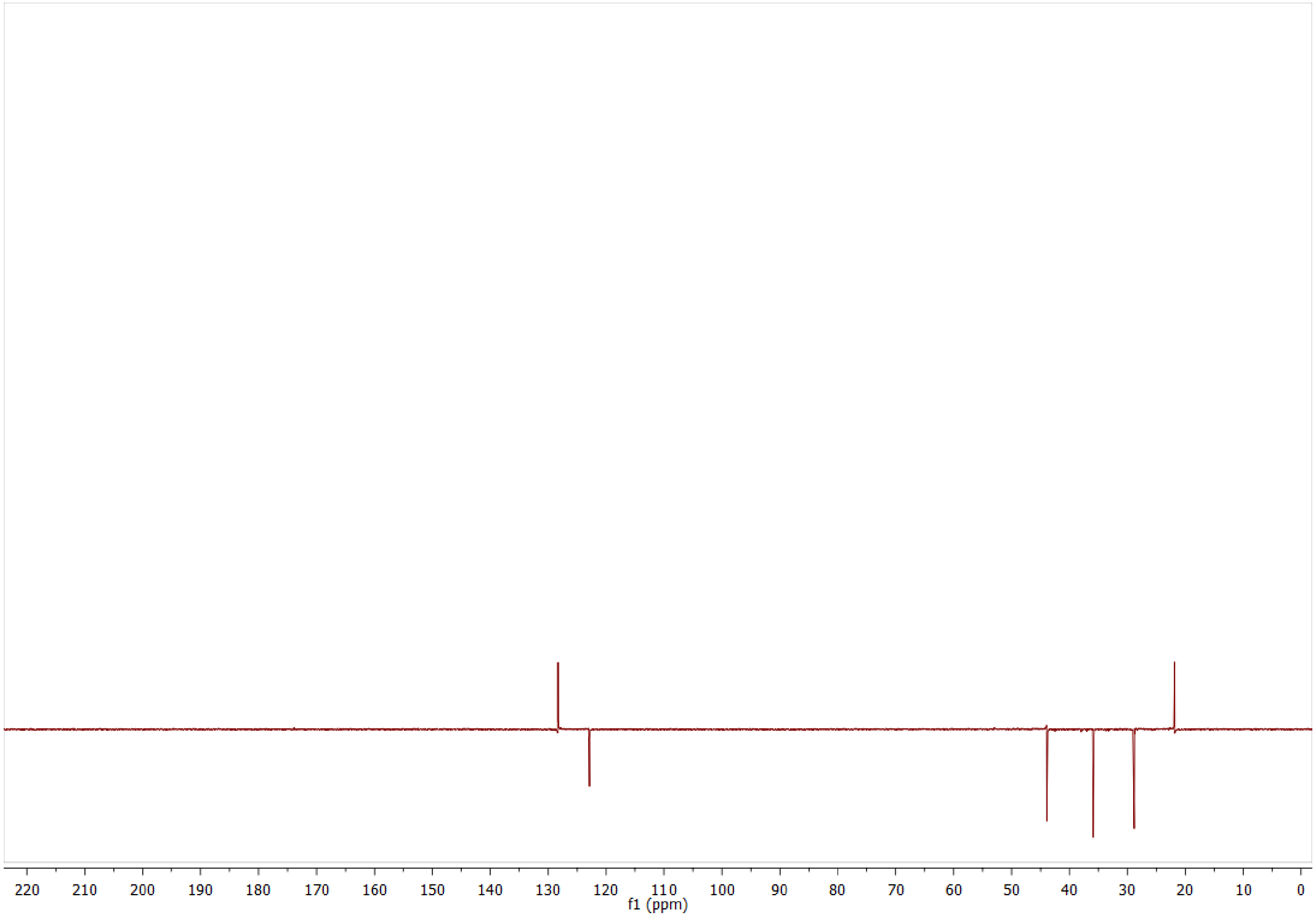

Figure S12: ^{13}C-DEPT spectrum (D_2O, 175 MHz) of AllMSP.

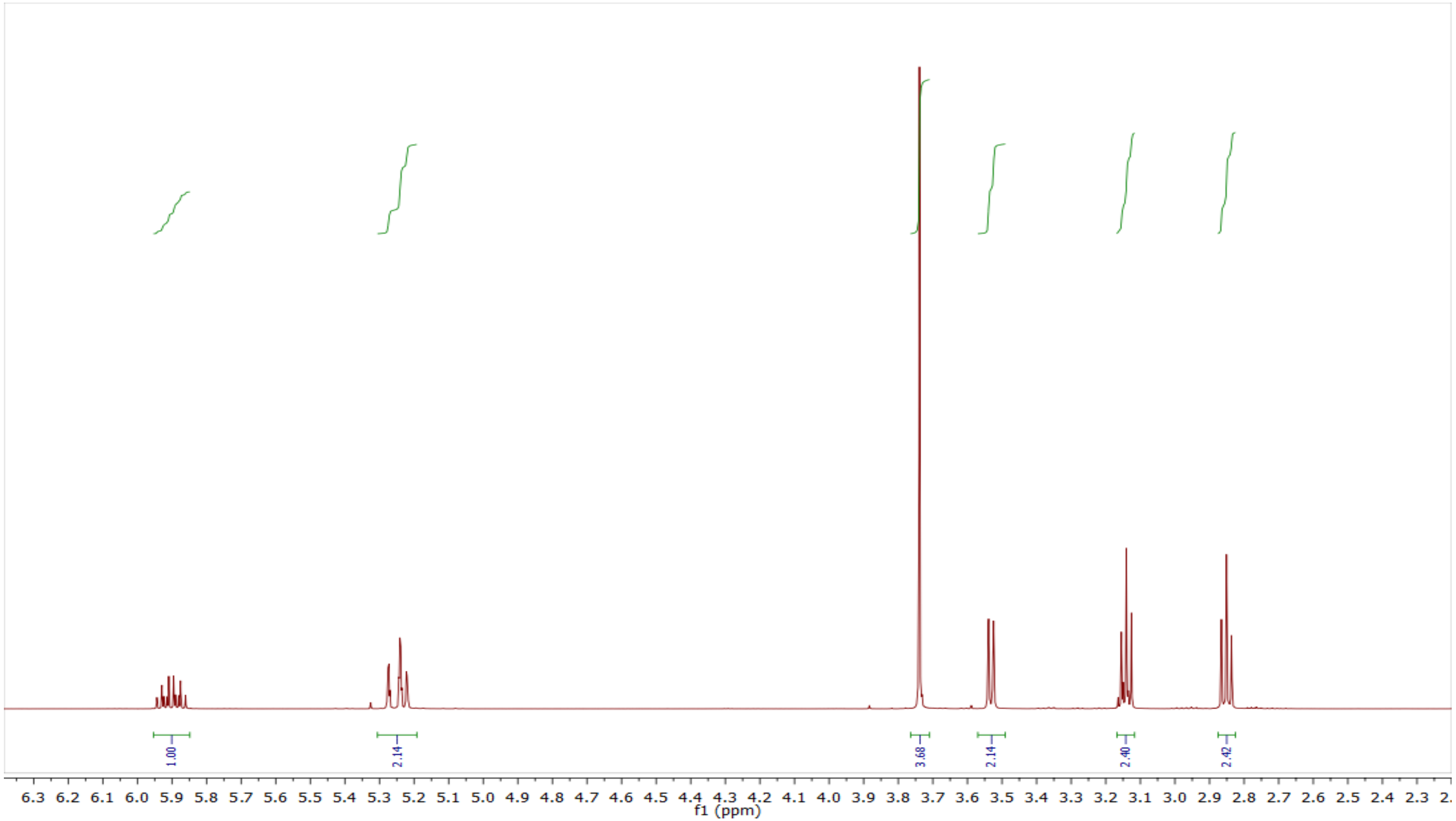

Figure S13: ^{1}H NMR spectrum ($CDCl_3$, 500 MHz) of **26**.

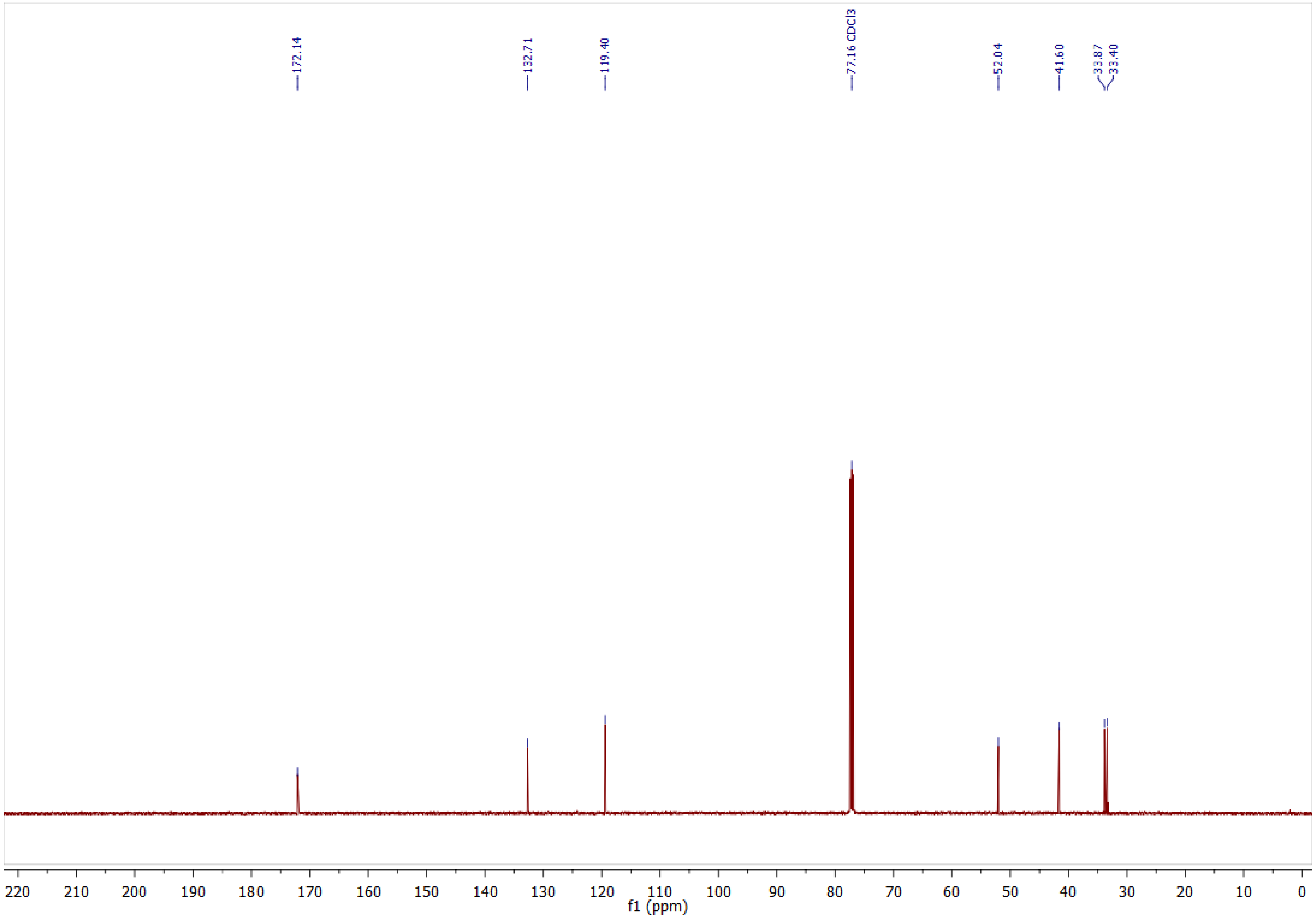

Figure S14: ^{13}C NMR spectrum ($CDCl_3$, 125 MHz) of **26**.

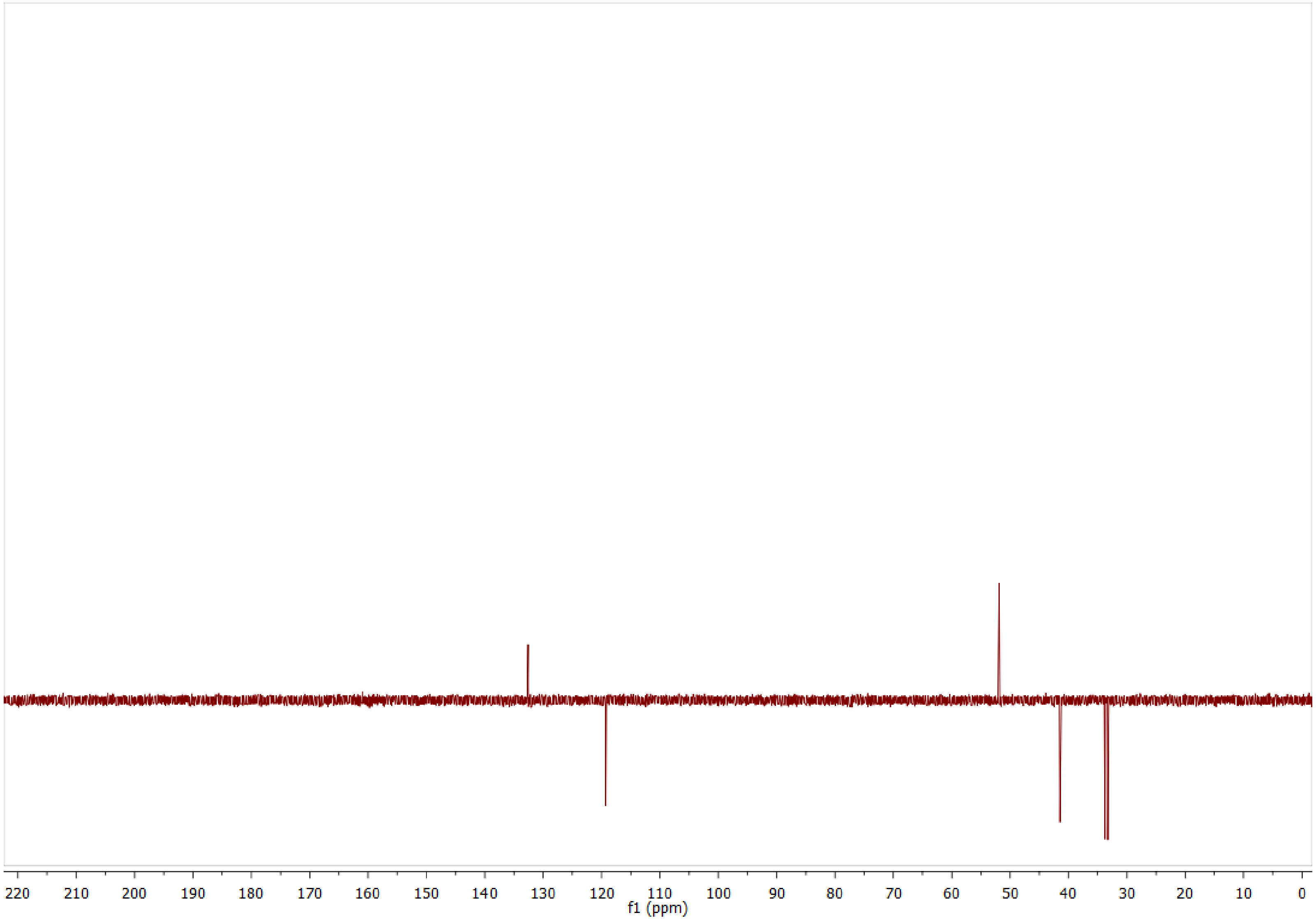

Figure S15: ^{13}C-DEPT spectrum ($CDCl_3$, 125 MHz) of **26**.

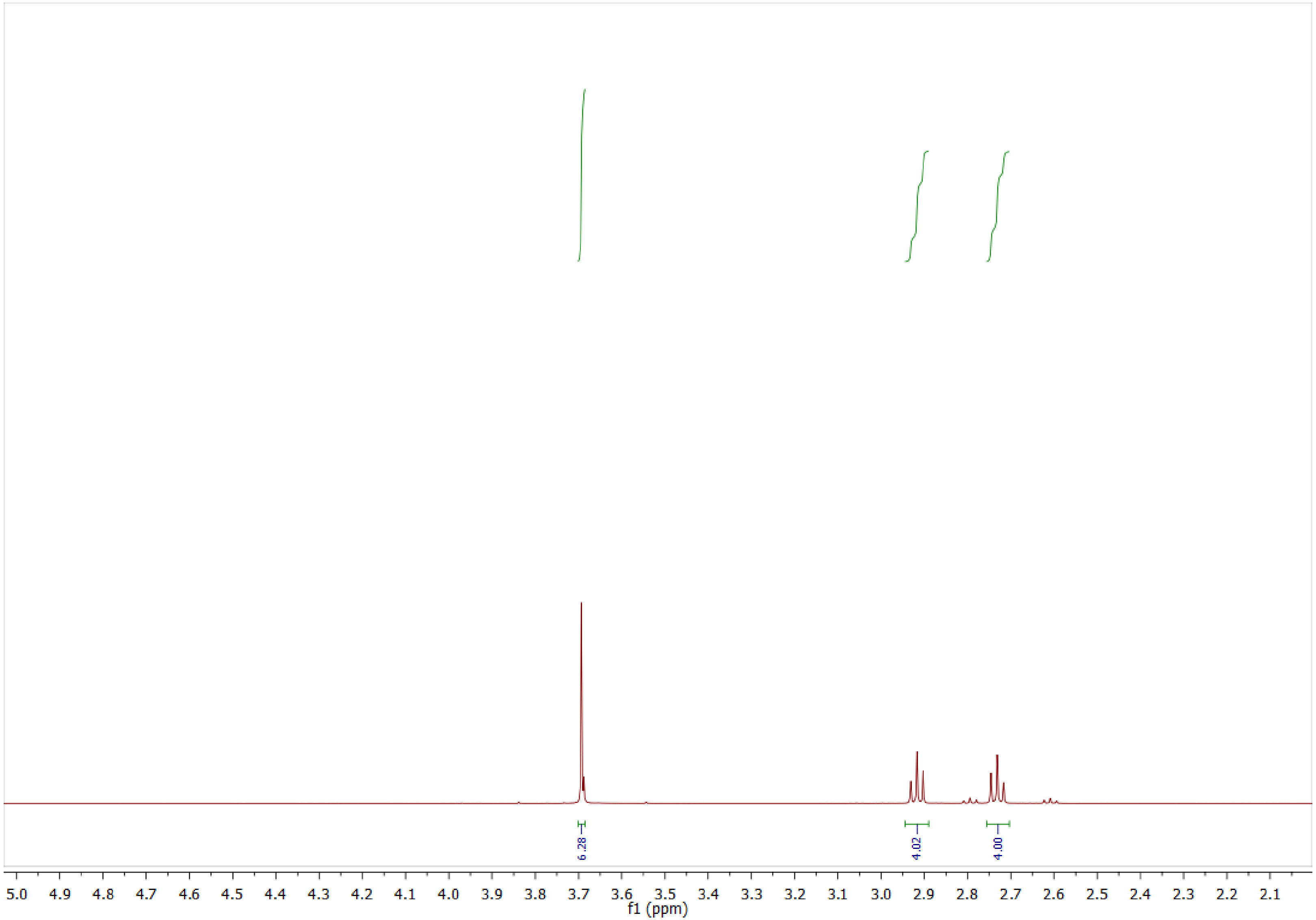

Figure S16: ^{1}H NMR spectrum ($CDCl_3$, 500 MHz) of **40**.

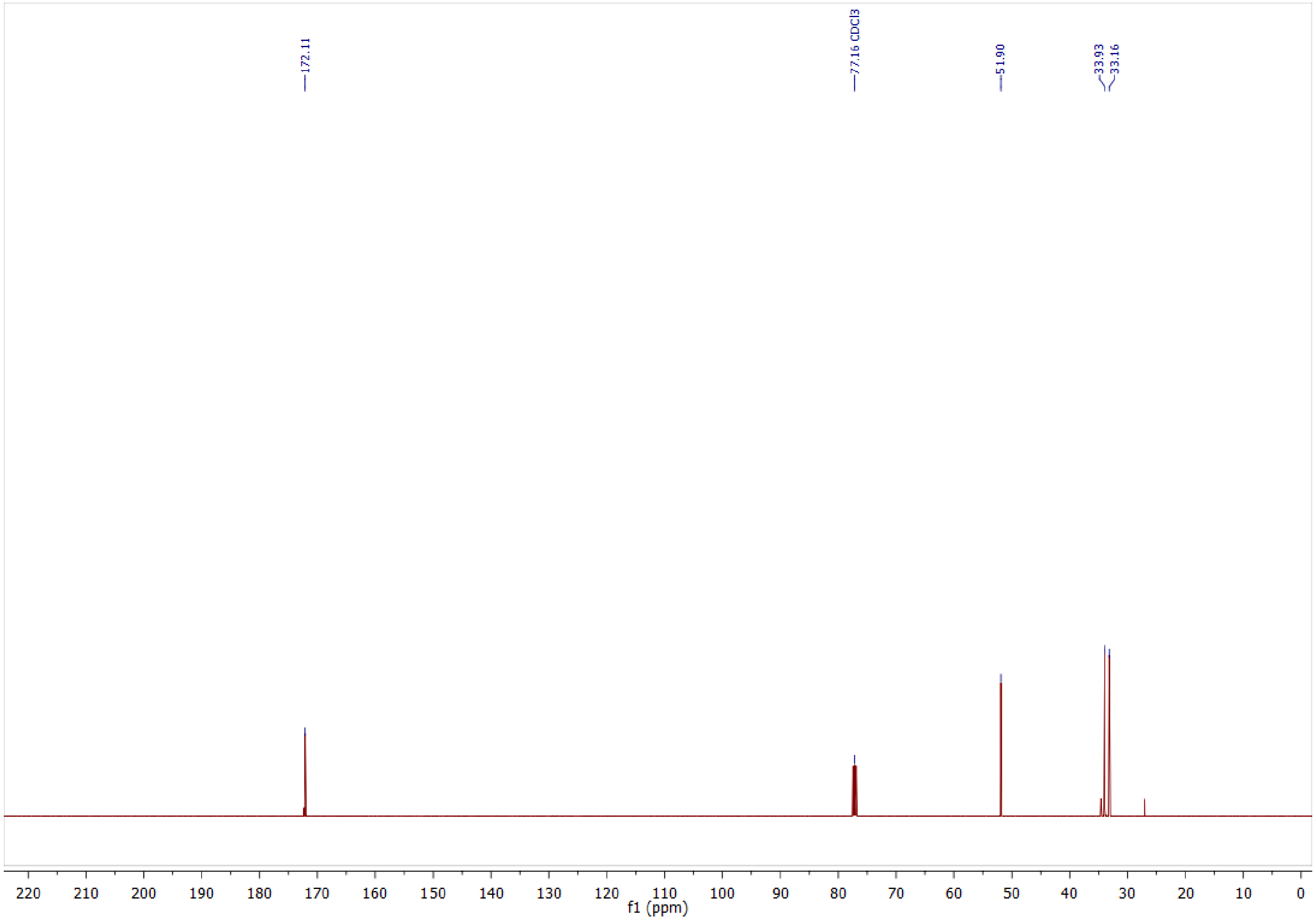

Figure S17: ^{13}C NMR spectrum ($CDCl_3$, 125 MHz) of **40**.

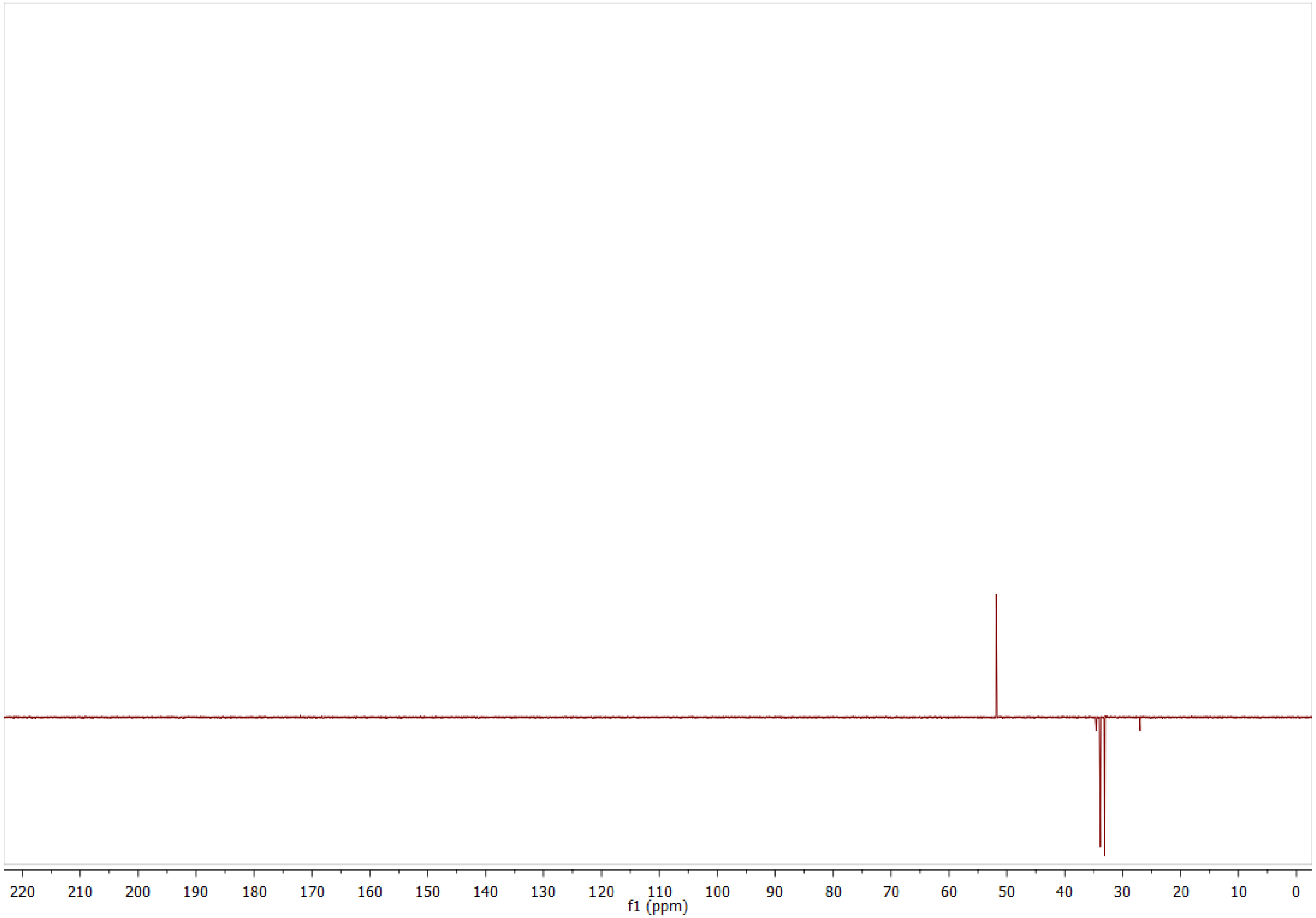

Figure S18: ^{13}C-DEPT spectrum ($CDCl_3$, 125 MHz) of **40**.

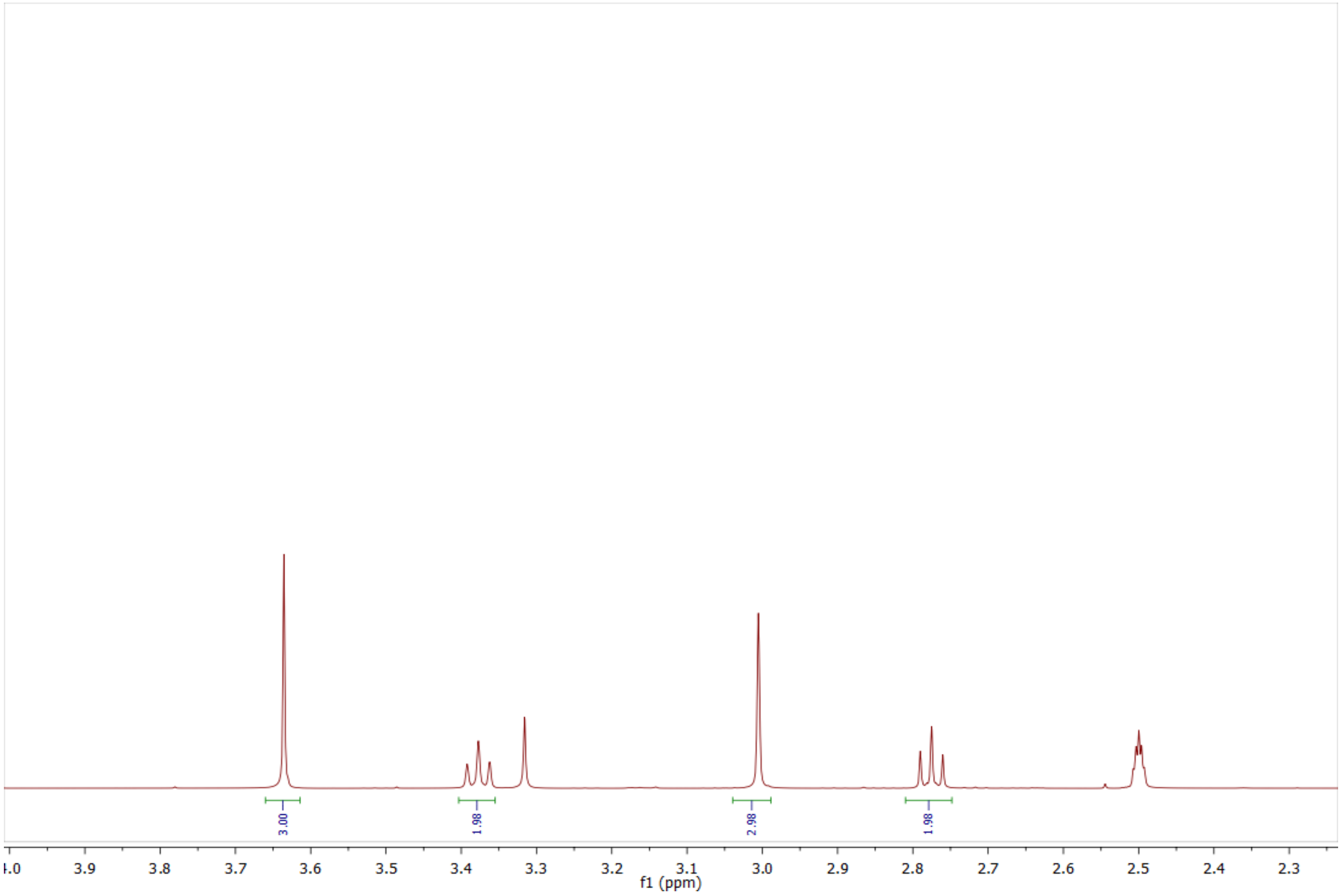

Figure S19: ^{1}H NMR spectrum (d_6-DMSO, 500 MHz) of **27**.

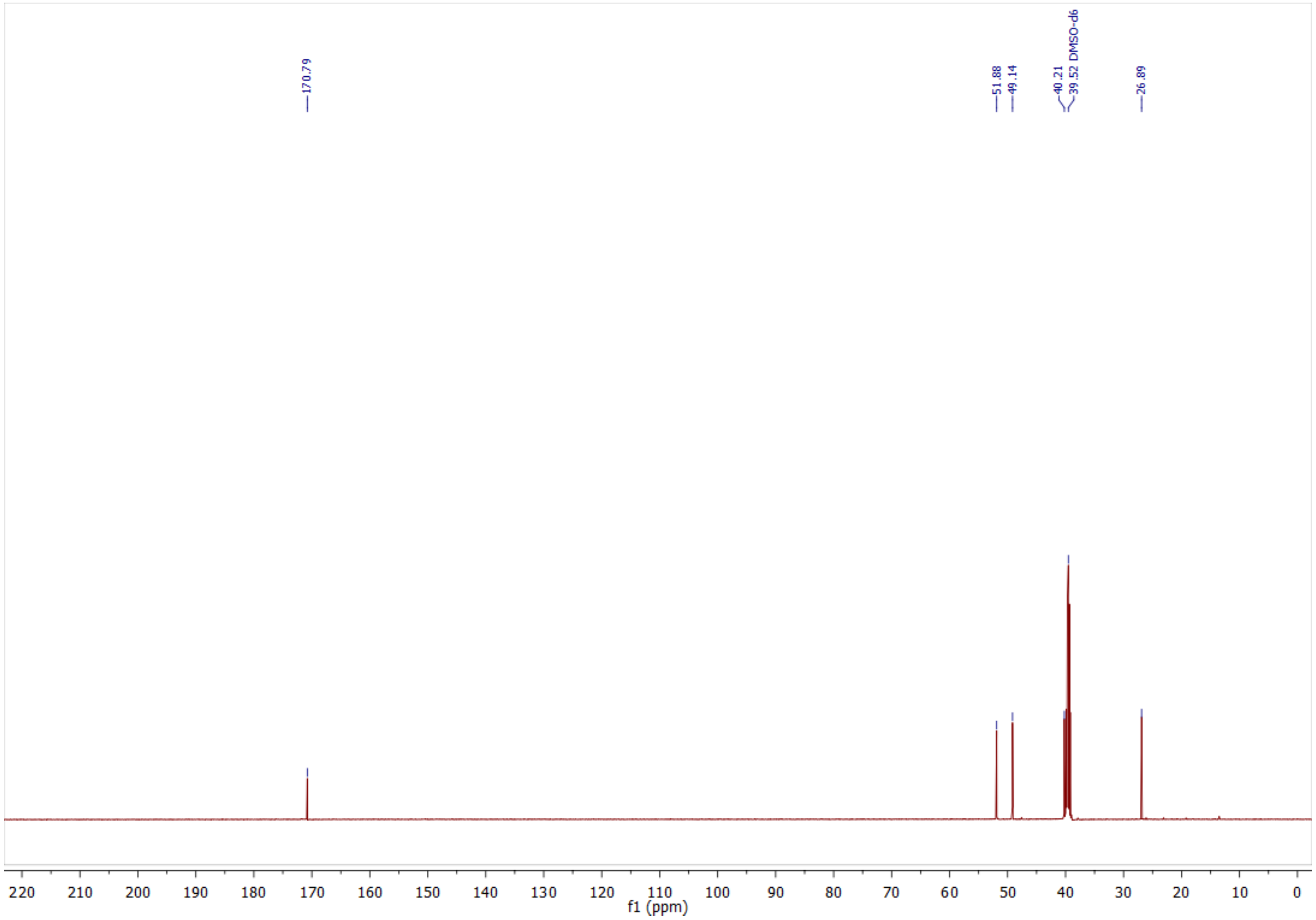

Figure S20: ^{13}C NMR spectrum (d_6-DMSO, 125 MHz) of **27**.

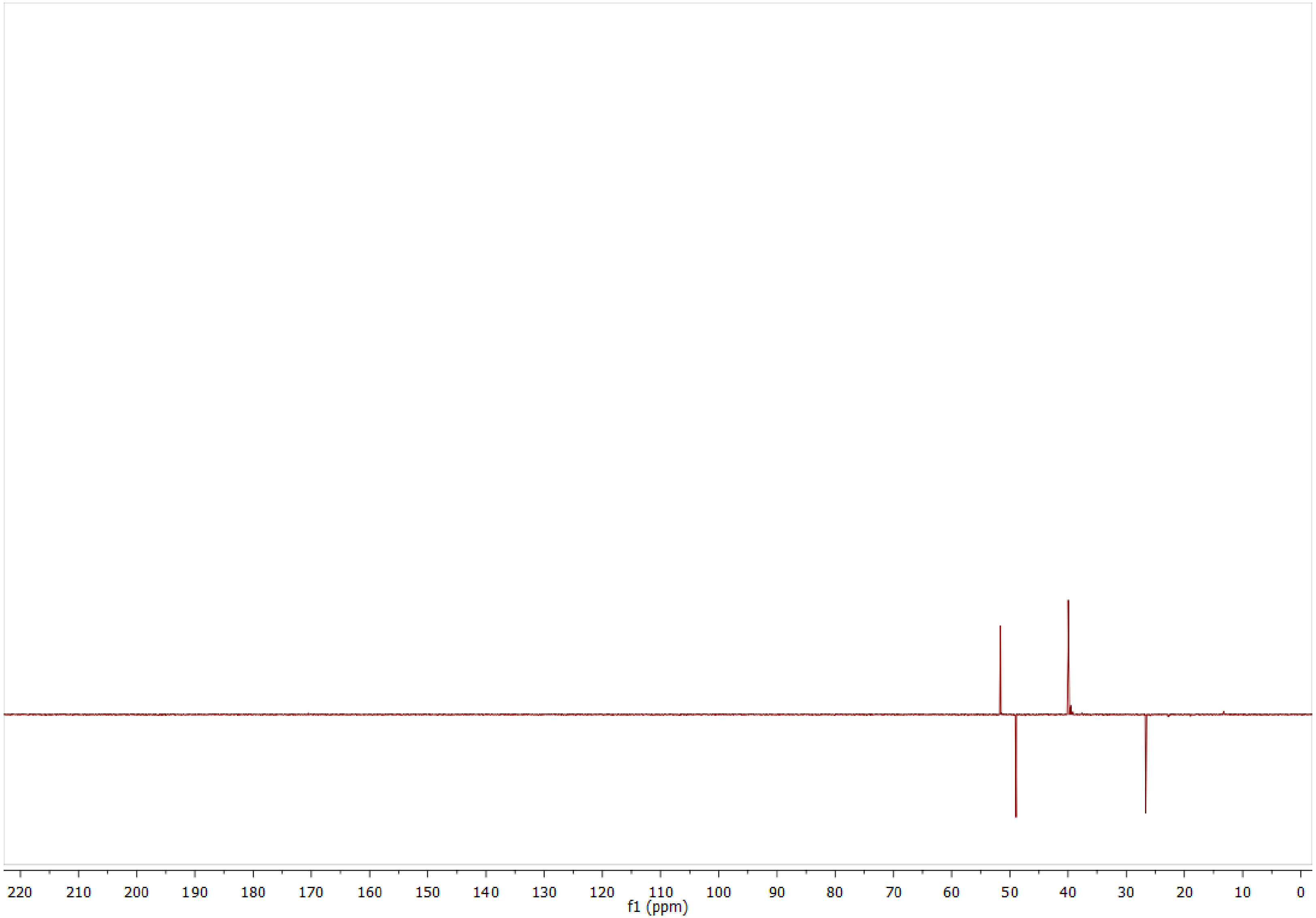

Figure S21: ^{13}C-DEPT spectrum (d_6-DMSO, 125 MHz) of **27**.
